# Predicting Long-Term Care Service Demands for Cancer Patients: A Machine Learning Approach

**DOI:** 10.3390/cancers15184598

**Published:** 2023-09-16

**Authors:** Shuo-Chen Chien, Yu-Hung Chang, Chia-Ming Yen, Ying-Erh Chen, Chia-Chun Liu, Yu-Ping Hsiao, Ping-Yen Yang, Hong-Ming Lin, Xing-Hua Lu, I-Chien Wu, Chih-Cheng Hsu, Hung-Yi Chiou, Ren-Hua Chung

**Affiliations:** 1 Institute of Population Health Sciences, National Health Research Institutes, Miaoli County 350, Taiwan; 2 National Center for Geriatrics and Welfare Research, National Health Research Institutes, Yunlin County 632, Taiwan; 3 Department of Risk Management and Insurance, Tamkang University, New Taipei City 251, Taiwan; 4 School of Public Health, College of Public Health, Taipei Medical University, Taipei 110, Taiwan

**Keywords:** machine learning, long-term care services, demands prediction, cancer patients, healthcare advancements, sensitivity analysis

## Abstract

**Simple Summary:**

Understanding the long-term care needs of cancer patients is crucial for healthcare providers and policymakers, as this area remains understudied. This research aims to fill this knowledge gap by employing machine learning algorithms to predict the kinds of services that these patients may require. We have developed two specialized models: one provides a generalized view of potential service needs, and the other makes more specific service-type predictions. Our findings identify not only the types of cancer that significantly differ in their care service usage but also key demographic and health-related factors that influence these needs. This research offers valuable insights that could guide the allocation of healthcare resources and customized care interventions for cancer patients.

**Abstract:**

Background: Long-term care (LTC) service demands among cancer patients are significantly understudied, leading to gaps in healthcare resource allocation and policymaking. Objective: This study aimed to predict LTC service demands for cancer patients and identify the crucial factors. Methods: 3333 cases of cancers were included. We further developed two specialized prediction models: a Unified Prediction Model (UPM) and a Category-Specific Prediction Model (CSPM). The UPM offered generalized forecasts by treating all services as identical, while the CSPM built individual predictive models for each specific service type. Sensitivity analysis was also conducted to find optimal usage cutoff points for determining the usage and non-usage cases. Results: Service usage differences in lung, liver, brain, and pancreatic cancers were significant. For the UPM, the top 20 performance model cutoff points were adopted, such as through Logistic Regression (LR), Quadratic Discriminant Analysis (QDA), and XGBoost (XGB), achieving an AUROC range of 0.707 to 0.728. The CSPM demonstrated performance with an AUROC ranging from 0.777 to 0.837 for the top five most frequently used services. The most critical predictive factors were the types of cancer, patients’ age and female caregivers, and specific health needs. Conclusion: The results of our study provide valuable information for healthcare decisions, resource allocation optimization, and personalized long-term care usage for cancer patients.

## 1. Introduction

Cancer is not only a leading cause of mortality worldwide but also a significant stressor on global healthcare systems [1]. The intricate nature of cancer, combined with often-debilitating treatments such as chemotherapy and radiation, results in a complex array of physical and psychosocial challenges for patients [2,3]. These challenges, which can significantly compromise a patient’s quality of life, underline the necessity of a multi-faceted approach to healthcare, one that goes beyond immediate medical treatment [4].

In this context, long-term care (LTC) services emerge as an essential component of comprehensive healthcare, particularly for the aging population and those grappling with chronic illnesses like cancer [3]. LTC services are multifaceted, aiming to address the needs of individuals who are hampered in their ability to manage daily living activities [5]. This includes not only medical tasks but also personal care and household chores, all contributing to the enhancement of the patient’s overall quality of life and health outcomes [6,7]. Nevertheless, with the ever-increasing demand for these indispensable services, optimizing the allocation of scarce LTC resources has become increasingly imperative [8].

Despite the critical role of LTC services in healthcare, the current literature is somewhat limited in delineating how these services are utilized specifically by cancer patients. Prior studies have employed machine learning (ML) techniques for predicting LTC needs; however, they often limit their scope to very specific service usage scenarios. For instance, two Japanese studies used healthcare insurance claims and multiclass classification to predict eligibility for government allowances instead of assessing demand for LTC services directly, with one study focusing on people over 75 [9,10]. In contrast, a study from Taiwan forecasts the scores for difficulties in Activities of Daily Living (ADL) and Instrumental Activities of Daily Living (IADL), achieving mean absolute errors of 17.67 and 1.31, respectively [11]. Meanwhile, another study focused on predicting the demand for emergency house call services, potentially neglecting other non-emergency but equally critical services [8]. Such a narrow focus inherently limits the generalizability of these methods, as it fails to capture the comprehensive care needs specific to cancer patients, who often require a variety of services due to the multi-faceted nature of their disease and treatment regimens [12,13]. A more nuanced understanding of the demand for LTC services among cancer patients, coupled with a tailored prediction model, is urgently needed to address these gaps in existing research.

To address these shortcomings and knowledge gaps, this study aims to provide a comprehensive and targeted overview of LTC service utilization patterns among cancer patients. First of all, we explored the costs and usage across a wide range of services and examined how these patterns differ among various forms of cancer. In addition, by employing machine learning (ML) techniques, we established both a comprehensive predictive model for long-term care (LTC) service utilization and specialized models for each specific service, thereby providing a flexible and universally applicable approach for understanding and anticipating LTC demands. Moreover, our study conducted a sensitivity analysis to identify the optimal usage frequency cutoff point that best distinguishes between those people who would and would not utilize LTC services.

## 2. Materials and Methods

This study is a retrospective analysis that employed data collected between August 2019 and December 2022 in Ping-Tung County, a regional administrative division in southern Taiwan. Ethical approval for this research was granted by the Institutional Review Board of the National Health Research Institutes, under protocol codes EC1091216-1 and 20211123.

### 2.1. LTC 2.0 Services

The study population for this research consists of individuals who have received services from LTC 2.0, a program formulated by the Taiwan central government and implemented by local authorities [14]. Before accessing LTC services, recipients undergo a comprehensive evaluation that includes various metrics such as demographics, disease history and condition, communication skills, short-term memory, Activities of Daily Living (ADL), Instrumental Activities of Daily Living (IADL), medical history, nutrition, and living environment [14,15]. The results of this assessment serve as variables for constructing our predictive model in this study. To identify cancer cases, we relied on evaluation records that indicated a cancer diagnosis within the past five years. Table S2 summarizes the services provided by LTC 2.0. There exist more than 50 distinct types, which can be basically classified into five categories, including homecare, daycare and adult foster service, professional service, transportation, respite service, and others [16].

### 2.2. Model Construction and Data Preprocessing

**Figure 1:**
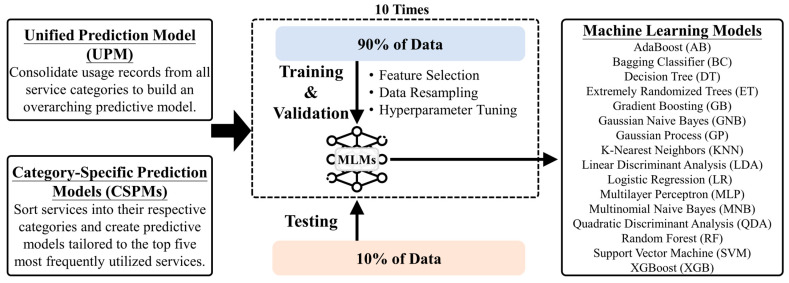
Conceptual framework for LTC service prediction model development.

Figure 1 illustrates the conceptual framework for our study’s long-term care (LTC) service demand prediction models. We developed two main types of predictive models for this research. The “Unified Prediction Model (UPM)” is a comprehensive model that takes into account all types of services available in the LTC system. In contrast, the “Category-Specific Prediction Models (CSPMs)” are specialized models focused on the five most frequently utilized types of services. Both models make use of a variety of machine learning methods in their formulation. The UPM aims for a broader, more generalized prediction, while the CSPMs offer more targeted forecasts for specific types of services.

In our dataset, we included a total of 462 features, of which 406 were related to the case (care recipient) and 56 were related to the caregiver (Table S3). In the data preprocessing phase, we streamlined the dataset by removing redundant features and converting numerical attributes into binary variables. To achieve this, we employed the median as a threshold for discretizing continuous variables, coding values greater than the median as one and those less than or equal to the median as zero. Regarding the treatment of missing values, we used the median to substitute the missing values for numerical types and introduced a new “Null” field to replace the missing state of a categorical feature. Following this preprocessing, we partitioned the dataset into training and testing subsets. To rigorously assess model performance, we executed 10-fold cross-validation solely on the training set.

To optimize model performance, several techniques were applied to the training dataset. Initially, the Least Absolute Shrinkage and Selection Operator (LASSO) was used for feature selection [17]. However, we found that in models for the top five most frequently used services, LASSO could remove all features at certain cutoff points. To mitigate this, we employed the Recursive Feature Elimination (RFE) method to select the 30 most important features [18]. Meanwhile, to address data imbalance, the Synthetic Minority Over-Sampling Technique (SMOTE) was applied [19]. Furthermore, the hyperparameter tuning was carried out using grid search methods, examining an average of approximately 50 different hyperparameters [20].

Finally, the top-performing model was selected using 10-fold cross-validation on the training dataset. We then evaluated and reported its performance on an independent test set that the model had never encountered before. We assessed the performance of all models using the Area Under the Receiver Operating Characteristic (AUROC) curve [21]. The AUROC serves as a measure of a model’s discriminatory ability, with performance classifications ranging from “no discrimination” (AUROC of 0.5) to “outstanding discrimination” (AUROC greater than 0.9) [21]. Additional evaluation metrics such as precision, recall, and F1 score were calculated using the Youden Index to provide a comprehensive understanding [22,23].

### 2.3. Sensitivity Analysis

Our objective is to predict whether a case will utilize LTC services. We defined usage status as either “usage” or “non-usage” based on the number of utilizations. To assess the impact of varying cutoff points on the model’s performance and to determine the optimal cutoff point that could effectively differentiate between usage and non-usage, we conducted a sensitivity analysis, while the conceptual framework is presented in Figure S2.

To conduct a thorough sensitivity analysis, we first organized all cases in ascending order according to the number of service users. We defined cutoff points at 0.5% percentile increments to frame this analysis. For each cutoff point, we deployed 16 distinct ML algorithms to construct predictive models.

In the case of the UPM, which considered various service categories as one, we designated a sensitivity analysis range of 15% to 85%. This selection was informed by the Pareto principle, commonly known as the 80–20 rule [24]. The principle suggests that approximately 80% of outcomes typically originate from just 20% of contributing factors. To ensure a more comprehensive data overview, we broadened this range by 5% at both the lower and upper bounds.

In contrast, when building CSPMs, we noted that the most frequently used service did not show any usage until the 75th percentile, accounting for less than a quarter of the total usage. Given this observation, we extended the upper limit of the sensitivity analysis range from 85% to 95%.

## 3. Results

During our study period from 2019 to 2022, we collected a total of 33,321 unique cases. Figure S3 illustrates the process of selecting the study cohort, which includes analyzing the disparities in LTC service utilization between cancer and non-cancer patients, as well as the development of a cancer-specific LTC service demand prediction ML model. Out of the total number of cases, 3333 patients were confirmed to have cancer based on their evaluation records, indicating a cancer diagnosis within the previous five years. Table S4 shows the demographics of all cases, as well as distinguishing between cancer and non-cancer cases. The entire dataset comprised 33,321 cases, including 3333 cancer cases and 29,988 non-cancer cases. The average age was marginally lower for cancer cases (74.88 years) compared to non-cancer cases (76 years), and there were more male cases in the cancer group (53.3%) than in the non-cancer group (41.6%). In terms of caregivers, those caring for cancer patients had a higher mean age (53.16 years) and were slightly less likely to be male (34.3%). Across all categories, 11.4% employed a caregiver, with fewer cancer cases (8.5%) doing so compared to non-cancer cases (11.7%). The statistical comparison of LTC service utilization between non-cancer and cancer cases can be found in the Supplementary Materials (Figure S1 and Table S1).

### 3.1. LTC Service Utilization Differences among Various Cancer Types

In Table 1, which elucidates the LTC comparative statistics on service use across various cancer types, a spectrum of noteworthy findings is presented (Figure S4). The table encompasses various metrics, including the number of total cases (N of cases), the number of service usages (N of usage), the T-statistic, and the *p*-value for each cancer type.

We employed a one-vs-all t-test to evaluate service usage differences among various types of cancer based on their number of service usages. The analysis revealed statistically significant deviations in service usage for four types of cancer: lung, liver, brain, and pancreatic. Specifically, lung cancer cases were marked by a mean usage of 135.9, supported by a significant result (t = −3.272, *p* = 0.001). Similarly, liver cancer had a mean usage of 123.7 (t = −2.831, *p* = 0.005). Conversely, brain cancer showed an elevated mean usage of 310.0 (t = 1.986, *p* = 0.047), indicative of higher service consumption. Pancreatic cancer also revealed significance, with a *p*-value of 0.015 (t = −2.448), and displayed a lower mean usage of 52.0.

Regarding usage rate, oral cancer cases were marked by a usage rate of 48.1, cervical by 47.2, other by 46.5, and myeloma by a strikingly lower rate of 19.5. These salient statistics provide not only a nuanced understanding of disparities in service usage rates among diverse cancer types but also insight into the usage rate. Such findings are pivotal for healthcare resource allocation and the formulation of more personalized patient care strategies.

**Figure 2:**
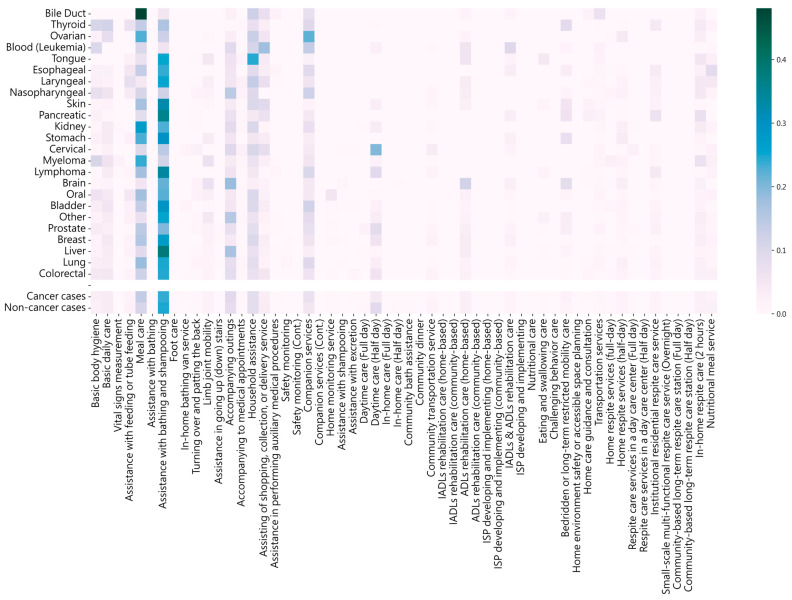
Number of LTC services used among different cancer types.

We examined the differences in LTC service usage among various types of cancers, and the results are visualized through the heat map in Figure 2. The X-axis labels represent the different cancer types sorted in ascending order based on the number of usages. The heat box for each cancer type was calculated by dividing the number of specific service usages by the total number of specific service usages.

The analysis of the utilization rates of different care services among various cancer types indicated distinct patterns. For instance, individuals diagnosed with pancreatic cancer showed a particularly high reliance (35.7% of total usage) on assistance for basic body hygiene. This elevated utilization rate can be attributed to the decreased mobility and energy levels of these patients due to the aggressive nature of pancreatic cancer and its treatment methods. Furthermore, a significant utilization rate of meal care services was observed among bile duct cancer cases, with nearly half of the total usages (48.1%), which could be attributed to their need for a specialized diet.

Notably, cases of blood cancer (Leukemia) and tongue cancer tended to utilize services such as household assistance (24.6% of total usage) and assistance with shopping, collection, or delivery service (17.7% of total usage) more frequently due to their communication and mobility skill disabilities. These analyses provide a detailed understanding of the care needs of different cancer patients and could inform strategies to optimize the provision of care services.

### 3.2. Top 20 Efficient Model Cutoff Points in UPM

We utilized 16 distinct machine learning algorithms, coupled with sensitivity analysis, to evaluate the influence of different cutoff thresholds on model performance and to pinpoint the optimal threshold for effectively distinguishing between usage and non-usage. The performance metrics were evaluated using an independent test set (Tables S5–S8). A comprehensive overview of the top 20 best performance model cutoff points is presented, ranked by their AUROC scores on the test set (Table 2), while additional details are available in Tables S5–S8. The models we assessed include Logistic Regression (LR), Linear Discriminant Analysis (LDA), Quadratic Discriminant Analysis (QDA), Bagging Classifier (BC), AdaBoost (AB), Extra Trees (ET), and XGBoost (XGB). To manage feature selection and class imbalance, we either employed LASSO as a standalone technique (denoted as “L”) or combined LASSO with SMOTE (denoted as “L + S”). The AUROC scores across these models ranged from 0.707 to 0.728.

The highest-performing model is the LR at COP = 84.0, which uses LASSO and SMOTE (L + S), achieving an AUROC of 0.728. This model also scores well in recall (0.740) and F1 score (0.411), with optimal parameters including a regularization parameter C = 10,000 and the solver ‘lbfgs’. Following closely is an LDA model at COP = 84.0, also using “L + S”, with an AUROC of 0.720, recall of 0.740, and an F1 score of 0.413. The LDA model is optimized with ‘lsqr’ as the solver and auto shrinkage.

Among the other standout models are a QDA at COP = 72.0 configuration using L + S with an AUROC of 0.718 and a particularly high recall rate of 0.808, and an XGBoost model at COP = 72.0 also using L + S, which attained an AUROC of 0.711 with an impressive recall of 0.859. These models also demonstrated strong F1 scores, at 0.495 and 0.480, respectively. In summary, the table showcases a well-rounded performance snapshot of the models across multiple evaluation metrics, indicating that varying combinations of hyperparameters and feature engineering techniques like LASSO and SMOTE can lead to different levels of model efficacy.

**Figure 3:**
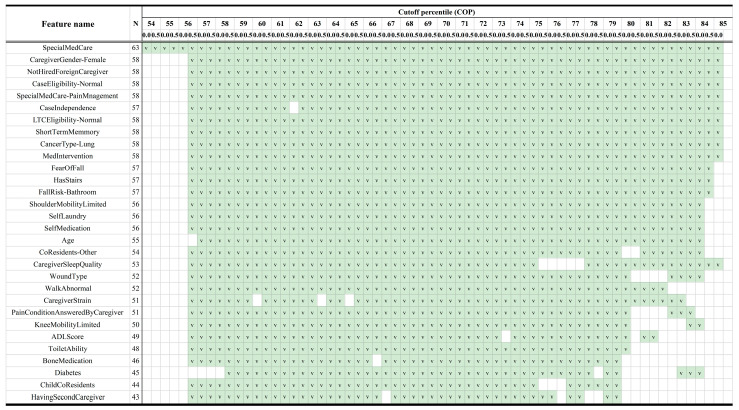
Top 30 most important features (N = number of cutoff points passed feature selection).

Figure 3 illustrates the top 30 most important features among the model cutoff points, and the number of cutoff points passed feature selection (N). The definition for each feature name is listed in Table S9. Under the main column ‘Cutoff Percentile (COP)’ labeled ‘54,’ there are two sub-columns: ‘0.0’ and ‘0.5’. These sub-columns represent the values 54.0 and 54.5, respectively, and this pattern continues in the same manner for subsequent values such as 55.0, 55.5, etc. Specialized medical care is highlighted by the prominence of features like SpecialMedCare and SpecialMedCare-PainManagement, suggesting the significance of tailored medical approaches in care needs. Physical limitations also play a substantial role, with features like KneeMobilityLimited and ShoulderMobilityLimited making it into the top ranks.

In the caregiving context, attributes related to the caregiver and household conditions are noteworthy. For instance, CaregiverGender-Female and HavingSecondCaregiver point to the caregiving landscape within the household, while features such as NotHiredForeignCaregiver and CoResidents-Other offer insights into the living circumstances. Moreover, CaregiverSleepQuality and CaregiverStrain indicate the emotional and physical toll on caregivers, emphasizing the necessity for supportive measures.

Finally, features focusing on the patient’s environment and their ability to manage daily activities are also crucial. These include FallRisk-Bathroom, SelfLaundry, and SelfMedication, which highlight the patient’s capability for self-care. Practical challenges in daily life are underlined by features like HasStairs and ToiletAbility.

### 3.3. Top 5 Efficient Model Cutoff Points in CSPM

Table 3 presents the top five performance metrics for model cutoff points in the Category-Specific Prediction Models (CSPMs) across the five most frequently utilized service categories: “Assistance with Bathing and Shampooing”, “Accompanying Outings”, “Meal Care”, “Household Assistance”, and “Companion Services”. The models are evaluated based on four key metrics: Area Under the Receiver Operating Characteristic Curve (AUROC), recall, precision, and F1 score. Additional parameters, including Cutoff Point (COP), Cutoff Value (COV), Number of Labels (NoLs), Number of Features (NoFs), and Best Parameters for each model, are also detailed in the table.

In the caregiving sector, the type of service and frequency of its use provided a context for model performance. For instance, the “Assistance with Bathing and Shampooing” service, utilized 61,451 times, saw the QDA model performing optimally at COP = 95.0 (AUROC = 0.837, recall = 0.929, precision = 0.108, F1 = 0.194). In comparison, the “Accompanying Outings” service, utilized 38,475 times, showed its best performance with the RF model at COP = 95.0 (AUROC = 0.841, recall = 0.857, precision = 0.130, F1 = 0.226).

For services with fewer instances, such as “Meal Care” and “Household Assistance”, utilized 37,279 and 35,928 times, respectively, different models took the lead. For “Meal Care”, the LR model was most effective at COP = 93.5 (AUROC = 0.784, Recall = 0.800, Precision = 0.134, F1 = 0.230). In “Household Assistance”, the BC model emerged as the best at COP = 90.0 (AUROC = 0.777, recall = 0.688, precision = 0.265, F1 = 0.383).

Finally, the “Companion Services”, used 34,154 times, was best modeled by QDA at COP = 94.0 (AUROC = 0.799, recall = 0.684, precision = 0.191, F1 = 0.299). The efficacy of machine learning models in these caregiving scenarios often varies depending on the service type and frequency of use, underlining the importance of the choice of cutoff points.

## 4. Discussion

### 4.1. Main Findings

In the current study, we have successfully engineered machine learning models and incorporated sensitivity analysis to forecast the LTC service needs of cancer patients. Few existing studies explore the demand prediction for LTC services, particularly for those afflicted with cancer. To the best of our knowledge, this is the first study providing a comprehensive solution by developing both unified and category-specific models to address this gap.

Statistical analysis results of 20 different types of cancer revealed significant variations in service usage. Specifically, lung, liver, brain, and pancreatic cancers showed statistically significant differences in service usage compared to other types of cancer. This suggests that the type of cancer may be a critical factor in determining LTC service needs.

Our sensitivity analysis pinpoints the optimal cutoff point for distinguishing between usage and non-usage cases with greater efficiency. As for the UPM, it achieved an AUROC of 0.728 at the 84.0 percentile cutoff, suggesting that 84% of patients are predicted not to require the service. Meanwhile, the CSPM demonstrated robust performance, achieving an AUROC ranging from 0.777 to 0.837 for predicting the top five most frequently utilized services.

In addition to these achievements, our results further illuminate the key determinants of LTC service demand among cancer patients. Factors such as demographics (e.g., patient age and female caregiver), health-related attributes (e.g., the need for specialized medical care, specific cancer types like lung cancer, and limited knee mobility), and caregiving context (e.g., caregiver strain and patient independence) emerged as crucial features influencing the utilization of LTC services among this patient population.

### 4.2. Implications

The predictive models formulated in this study offer immediately actionable insights for healthcare providers and policymakers [25]. Medical professionals can employ these algorithms for precise risk stratification, allowing them to pinpoint patients who are at higher risk of needing long-term care (LTC) services. Such targeted identification facilitates optimized resource allocation and timely interventions [26]. In the context of Taiwan, government agencies could specifically focus on individuals who have not yet utilized LTC services but are identified as potential users by the model [14]. These individuals might face barriers such as financial constraints or geographic isolation, making it challenging to access necessary services. To address this, the government could devise specialized LTC plans tailored to their unique needs. Concurrently, policymakers can leverage the predictive models to make informed decisions concerning healthcare planning [8]. This could involve evaluating the necessity of expanded LTC facilities or specialized services in regions showing a high incidence of particular types of cancer.

Like the previous research study on informal caregiver burden, the role of female caregivers remains a significant predictive factor in demographic considerations, reflecting the ongoing predominance of women in caregiving roles [27,28]. Health-related aspects of the patients, such as specialized medical needs, mobility limitations, and the ability to perform daily living activities independently, constitute important variables [16,29]. These needs are precisely what long-term care (LTC) services aim to address [16]. Notably, factors related to the caregiver—such as sleep quality, caregiving burden, and the absence of professional nursing care—also emerge as significant predictors [30,31]. These data suggest that the likelihood of utilizing LTC services increases when caregivers face higher levels of caregiving burden.

Drawing on our findings, we propose that future research should consider the following factors while designing analogous studies. First, individual-level prediction models should be adopted [32]. These would allow for customized, unique care plans for patients, considering their health status, lifestyle, and personal preferences. Second, including a more varied array of features, such as clinical data, financial status, living conditions, family support, and exercise status, is crucial [33]. These factors markedly influence the utilization of LTC services. Therefore, their integration would amplify the predictive power of the model. Lastly, we recommend an analysis of the impact of these services on disease progression, considering their potential to either hasten or decelerate deterioration [34]. By comprehending these relationships, we can strategically plan and deliver care interventions. This approach optimizes resource usage and improves patient outcomes. The insights derived from this study could offer valuable insights for researchers and policymakers to distribute the limited LTC resources in the future effectively.

### 4.3. Comparison with Previous Research

Previous research has largely overlooked the prediction of long-term care needs, specifically for cancer patients, focusing more on evaluating the likelihood of individuals qualifying for government financial subsidies. For example, Sato J.’s study leveraged historical healthcare insurance claims data to build a predictive model using multiclass classification and a gradient-boosting decision tree, achieving a high level of accuracy with a weighted average precision of 0.872, recall of 0.878, and an F-value of 0.873 [9]. Simultaneously, the study conducted by H. Fukunishi also utilized the same dataset but concentrated on predicting the needs of individuals aged 75 and older. The study achieved a precision score of 0.694 and a recall score of 0.505 [10]. Our own model also yielded promising results, achieving an AUROC of 0.728 using Logistic Regression techniques. However, the performance was limited by data imbalance, with the best F1 score reaching only 0.572 when employing the XGB algorithm. Interestingly, while all three studies identified age and sex to be significant factors in predicting long-term care needs, the research conducted by Sato J. and H. Fukunishi emphasized current health status as the most critical feature. In our model, although we included features relating to eligibility levels, they did not prove to be as crucial. This underscores the existence of other significant determinants for cancer patients in deciding whether to utilize long-term care services.

In another study, Sun Y. used medical and long-term care claims data to predict who among the elderly who regularly receive home visits are likely to require frequent emergency house calls [8]. The performance of their study closely aligns with ours: When using all 19 variables for prediction, they achieved an AUROC (Area Under the Receiver Operating Characteristic Curve) of 0.734. When making rule-based predictions using only the three most critical factors, the AUROC was 0.707. Notably, these key factors include home oxygen therapy, long-term care need level, and cancer. In our research, we examine home oxygen therapy as a component of Special Medical Care (SpecialMedCare), which is an important feature contributing to our predictions. Specifically, we delve deeper into variations in its usage among different types of cancer. We find that lung, liver, brain, and pancreatic cancers exhibit distinct patterns, setting our study apart from others in the field. Meanwhile, K.M. Chen’s model uses numerical output to forecast future usage frequency, which differs from previous models that relied on binary or multi-class classification. This offers a new thinking framework for consideration in subsequent research [11].

### 4.4. Limitations

There are several limitations to this study. First, in our research, we used the summation of usage to calculate and determine whether LTC services would be utilized. However, we neglected to consider the time series characteristics, which could have provided valuable insights. Second, relying on retrospective data may introduce selection bias, potentially affecting the accuracy of our prediction models. Lastly, our study only recruited cases from a specific county in the southern part of Taiwan, limiting the generalizability of our findings to other populations or healthcare systems.

## 5. Conclusions

This study offers a groundbreaking approach by using machine learning models to forecast LTC service needs among cancer patients, a significantly understudied subject. By identifying crucial variables like cancer type and demographic factors, our models pave the way for more personalized care and improved patient outcomes. These predictive tools not only showcase robust performance but also present actionable insights for better resource allocation and policymaking. Like previous works, this research underscores the transformative potential of data-driven models in healthcare, and it sets the stage for future studies to refine these tools for broader LTC scenarios.

## Figures and Tables

**Table 1:** LTC comparative statistics on service use in various cancer cases.

Cancer Types	N of Cases			N of Usage		T-Statistic	*p*-Value
	Total	Users	%	Mean	(SD)		
Colorectal	586	236	40.3	216.5	(327.0)	0.465	0.642
Lung	541	192	35.5	135.9	(276.1)	−3.272 ***	0.001
Liver	334	112	33.5	123.7	(187.4)	−2.831 **	0.005
Breast	330	138	41.8	258.2	(363.4)	1.920	0.055
Prostate	313	132	42.2	214.7	(314.9)	0.267	0.789
Other	187	87	46.5	187.9	(309.3)	−0.576	0.565
Bladder	176	66	37.5	264.1	(294.4)	1.440	0.150
Oral	158	76	48.1	271.8	(357.7)	1.762	0.078
Brain	124	39	31.5	310.0	(405.1)	1.986 *	0.047
Lymphoma	119	39	32.8	237.3	(345.8)	0.577	0.564
Myeloma	113	22	19.5	248.4	(584.0)	0.591	0.555
Cervical	106	50	47.2	259.2	(410.7)	1.137	0.256
Stomach	104	35	33.7	185.1	(253.4)	−0.408	0.683
Kidney	84	27	32.1	270.1	(425.0)	1.004	0.316
Pancreatic	67	26	38.8	52.0	(76.2)	−2.448 *	0.015
Skin	61	25	41.0	205.8	(361.2)	−0.025	0.980
Nasopharyngeal	58	25	43.1	222.4	(467.8)	0.231	0.817
Laryngeal	58	21	36.2	249.4	(361.0)	0.592	0.554
Esophageal	55	18	32.7	210.7	(214.7)	0.042	0.967
Tongue	54	25	46.3	180.2	(271.3)	−0.419	0.675

N = Number; SD = Standard deviation. * = *p* < 0.05; ** = *p* < 0.01; *** = *p* < 0.001.

**Table 2:** Summary of top 20 efficient performance model cutoff points (sorted by AUROC).

COP	COV	NoLs		NoFs	MLM		Performance Metrics					Best Parameters
							AUROC		Recall	Precision	F1	
		0	1				Test	Valid				
84	167.9	284	50	22	LR	(L + S)	0.728	0.690 (0.018)	0.74	0.285	0.411	C = 10,000, Sol = ‘lbfgs’
84	167.9	284	50	22	LDA	(L + S)	0.72	0.687 (0.026)	0.74	0.287	0.413	NC = None, S = ‘auto’, Sol = ‘lsqr’
72	37	256	78	39	QDA	(L + S)	0.718	0.658 (0.034)	0.808	0.403	0.495	RP = 0.2, SC = True
78	80	271	63	38	BC	(L + S)	0.714	0.639 (0.039)	0.825	0.297	0.437	MF = 0.5, MS = 0.5, NE = 100
84	167.9	284	50	22	AB	(L + S)	0.713	0.691 (0.048)	0.74	0.28	0.407	LR = 1, NE = 200
70.5	32	249	85	37	QDA	(L + S)	0.713	0.657 (0.032)	0.812	0.365	0.504	RP = 0.3, SC = True
71.5	36	254	80	59	QDA	(L + S)	0.711	0.666 (0.032)	0.713	0.385	0.5	RP = 0.2, SC = True
72.5	40	259	75	38	ET	(L + S)	0.711	0.649 (0.035)	0.827	0.348	0.49	MD = 10, MSS = 5, NE = 200
84	167.9	284	50	22	QDA	(L + S)	0.711	0.675 (0.034)	0.78	0.267	0.398	RP = 0.2, SC = True
72	37	256	78	39	XGB	(L + S)	0.711	0.641 (0.030)	0.859	0.333	0.48	G = 0.1, LR = 0.05, MD = 8, MCW = 2, NE = 1000
72.5	40	259	75	38	BC	(L + S)	0.71	0.644 (0.036)	0.747	0.358	0.477	MF = 0.5, MS = 1.0, NE = 100
83	150	282	52	40	QDA	(L + S)	0.71	0.676 (0.044)	0.808	0.278	0.414	RP = 0.2, SC = True
83	150	282	52	40	AB	(L + S)	0.71	0.687 (0.028)	0.75	0.293	0.422	LR = 1, NE = 200
69.5	29	243	91	36	QDA	(L + S)	0.71	0.664 (0.040)	0.736	0.435	0.547	RP = 0.4, SC = True
66.5	20.8	232	102	56	XGB	(L + S)	0.71	0.630 (0.040)	0.873	0.426	0.572	G = 0.1, LR = 0.05, MD = 8, MCW = 2, NE = 1000
72.5	40	259	75	38	AB	(L + S)	0.709	0.682 (0.034)	0.653	0.353	0.458	LR = 1, NE = 200
83.5	157.2	282	52	44	LDA	(L + S)	0.709	0.683 (0.031)	0.712	0.266	0.387	NC = None, S = ‘auto’, Sol = ‘lsqr’
70.5	32	249	85	37	XGB	(L + S)	0.708	0.644 (0.025)	0.682	0.406	0.509	G = 0.1, LR = 0.05, MD = 8, MCW = 3, NE = 1000
84	167.9	284	50	22	LR	(L)	0.708	0.688 (0.033)	0.86	0.231	0.364	C = 207, Sol = ‘newton-cg’
68	24	236	98	37	QDA	(L + S)	0.707	0.662 (0.032)	0.643	0.46	0.536	RP = 0.3, SC = True

COP = Cutoff Point; COV= Cutoff Value; NoLs = Number of Labels; NoFs = Number of Features; MLM = Machine Learning Models; AUROC = Area Under the Receiver Operating Characteristic; LR = Learning Rate; NE = Number of Estimators; MF = Max Features; MS = Max Samples; MD = Max Depth; MSS = Min Samples Split; NC = Number of Components; S = Shrinkage; Sol = Solver; C = Regularization Parameter; RP = Regularization Parameter; SC = Store Covariance; G = Gamma; MCW = Min Child Weight. The optimal point was determined using the Youden index; the valid column of AUROC represents the average 10-fold cross-validation scores, with the standard deviation given in parentheses.

**Table 3:** Top 5 model cutoff points in five most frequently used service categories.

COP	COV	NoLs		NoFs	MLM		Performance Metrics					Best Parameters
							AUROC		Recall	Precision	F1	
		0	1				Test	Valid				
**Assistance with bathing and shampooing (number of usages = 61,451)**												
95	129.8	320	14	30	QDA	(RFE)	0.837	0.764 (0.043)	0.929	0.108	0.194	RP = 0.3, SC = True
95	129.8	320	14	30	LDA	(RFE)	0.826	0.773 (0.064)	1	0.091	0.167	S = 0.1, Sol = ‘lsqr’
95	129.8	320	14	30	LR	(RFE)	0.824	0.767 (0.070)	0.857	0.118	0.207	C = 100, Sol = ‘newton-cg’
95	129.8	320	14	30	GB	(RFE)	0.806	0.751 (0.037)	0.929	0.103	0.186	MIP = 50, NRO = 0
93.5	97	314	20	30	LR	(RFE)	0.795	0.750 (0.042)	0.8	0.151	0.254	C = 10,000, Sol = ‘newton-cg’
**Accompanying outings (number of usages = 38,475)**												
95	53	320	14	30	RF	(RFE)	0.841	0.668 (0.050)	0.857	0.13	0.226	B = True, MD = 10, MSS = 5, NE = 200
95	53	320	14	30	QDA	(RFE)	0.815	0.687 (0.054)	0.857	0.1	0.179	RP = 0.1, SC = True
95	53	320	14	30	GB	(RFE)	0.809	0.675 (0.045)	0.857	0.111	0.197	LR = 0.1, MD = 3, NE = 100
95	53	320	14	30	XGB	(RFE)	0.798	0.657 (0.072)	0.929	0.089	0.163	G = 0.1, LR = 0.05, MD = 8, MCW = 2, NE = 300, SS = 0.7
94	39	315	19	30	GNB	(RFE)	0.786	0.667 (0.056)	0.789	0.169	0.278	VS = 1 × 10^−9^
**Meal care (number of usages = 37,279)**												
93.5	19	314	20	30	LR	(RFE)	0.784	0.724 (0.073)	0.8	0.134	0.23	C = 1, Sol = ‘newton-cg’
93.5	19	314	20	30	LDA	(RFE)	0.779	0.728 (0.075)	0.75	0.15	0.25	S = None, Sol = ‘svd’
93.5	19	314	20	30	GB	(RFE)	0.773	0.705 (0.048)	0.75	0.139	0.234	MIP = 50, NRO = 0
94	26.1	315	19	30	LR	(RFE)	0.76	0.740 (0.048)	0.737	0.118	0.203	C = 100, Sol = ‘newton-cg’
93.5	19	314	20	30	GNB	(RFE)	0.76	0.674 (0.090)	0.8	0.131	0.225	VS = 1 × 10^−8^
**Household assistance (number of usages = 35,928)**												
90	26	302	32	30	BC	(RFE)	0.777	0.687 (0.045)	0.688	0.265	0.383	MS = 0.5, NE = 50
92	43.4	308	26	30	LDA	(RFE)	0.776	0.746 (0.044)	0.769	0.22	0.342	S = ‘auto’, Sol = ‘lsqr’
91	33	304	30	30	LR	(RFE)	0.765	0.745 (0.043)	0.767	0.207	0.326	C = 1, Sol = ‘newton-cg’
93.5	60.4	314	20	30	BC	(RFE)	0.765	0.677 (0.051)	0.9	0.133	0.232	MS = 1.0, NE = 50
89.5	24	301	33	30	SVM	(RFE)	0.76	0.628 (0.060)	0.848	0.19	0.311	C = 0.1, G = 1, K = ‘linear’
**Companion services (number of usages = 34,154)**												
94	26.1	315	19	30	QDA	(RFE)	0.799	0.646 (0.037)	0.684	0.191	0.299	RP = 0.1, SC = True
92.5	12	310	24	30	LDA	(RFE)	0.792	0.698 (0.077)	0.875	0.181	0.3	S = None, Sol = ‘svd’
94.5	33.5	320	14	30	MNB	(RFE)	0.79	0.686 (0.077)	0.857	0.099	0.178	A = 1
94.5	33.5	320	14	30	QDA	(RFE)	0.789	0.674 (0.082)	0.929	0.109	0.195	RP = 0.1, SC = True
92.5	12	310	24	30	LR	(RFE)	0.789	0.699 (0.049)	0.833	0.161	0.27	C = 10,000, Sol = ‘newton-cg’

COP = Cutoff Point; COV= Cutoff Value; NoLs = Number of Labels; NoFs = Number of Features; MLM = Machine Learning Models; AUROC = Area Under the Receiver Operating Characteristic; RP = reg_param; SC = store_covariance; S = shrinkage; Sol = solver; C = C; MIP = max_iter_predict; NRO = n_restarts_optimizer; B = bootstrap; MD = max_depth; MSS = min_samples_split; NE = n_estimators; LR = learning_rate; G = gamma; MCW = min_child_weight; SS = subsample; VS = var_smoothing; MS = max_samples; A = alpha; K = kernel. The optimal point was determined using the Youden index; the valid column of AUROC represents the average 10-fold cross-validation scores, with the standard deviation given in parentheses.

## Data Availability

The data supporting the findings of this study can be obtained from the Department of Long-Term Care Ping-Tung Government, but access restrictions apply due to licensing agreements. Therefore, these data are not publicly accessible. However, upon reasonable request and with permission from the Department of Long-Term Care Ping-Tung Government, the authors can provide access to the data.

## Supplementary Materials

The following supporting information can be downloaded at: https://www.mdpi.com/article/10.3390/cancers15184598/s1, Figure S1: Percentage utilization of LTC services among cancer and non-cancer cases. Figure S2: Conceptualization of sensitivity analysis. Figure S3: Cohort selection process in our study. Figure S4: Numbers of usages and costs for LTC services among cancer cases. Table S1: Statistical comparison between cases of non-/cancer with LTC service utilization. Table S2: Summary of service categories provided by Taiwanese LTC 2.0. Table S3: Features used in this study. Table S4: Demographics of the datasets. Table S5: Results of sensitivity analysis (performance measured by AUROC). Table S6: Results of sensitivity analysis (performance measured by Recall). Table S7: Results of sensitivity analysis (performance measured by Precision). Table S8: Results of sensitivity analysis (performance measured by F1-Score). Table S9: Definitions of feature names.
