# Supplementary material for: Predicting Long-Term Care Service Demands for Cancer Patients: A Machine Learning Approach
Source: Cancers (Basel). 2023 Sep 16;15(18):4598. doi: 10.3390/cancers15184598 (PMC10526410; doi:10.3390/cancers15184598)
Supplement: Supplementary file 1 [file cancers-15-04598-s001.zip › cancers-2561795-supplementary.pdf]

Supplementary Materials

The LTC service utilization difference among non-cancer and cancer cases

Figure S1 shows the utilization rates of LTC services among both cancer and non-cancer cases. The bar graph shows the usage percentages for each category: cancer (represented in red) and non-cancer (represented in blue). Each bar's height corresponds to the proportion of specific LTC service usage in either non-cancer or cancer cases, which is calculated by dividing the count of a particular LTC service usage by the total count of LTC service usage within that category. On the X-axis, a label in red indicates that the usage percentage of that specific LTC service by cancer cases is greater than that by non-cancer cases, while a blue label denotes the opposite.

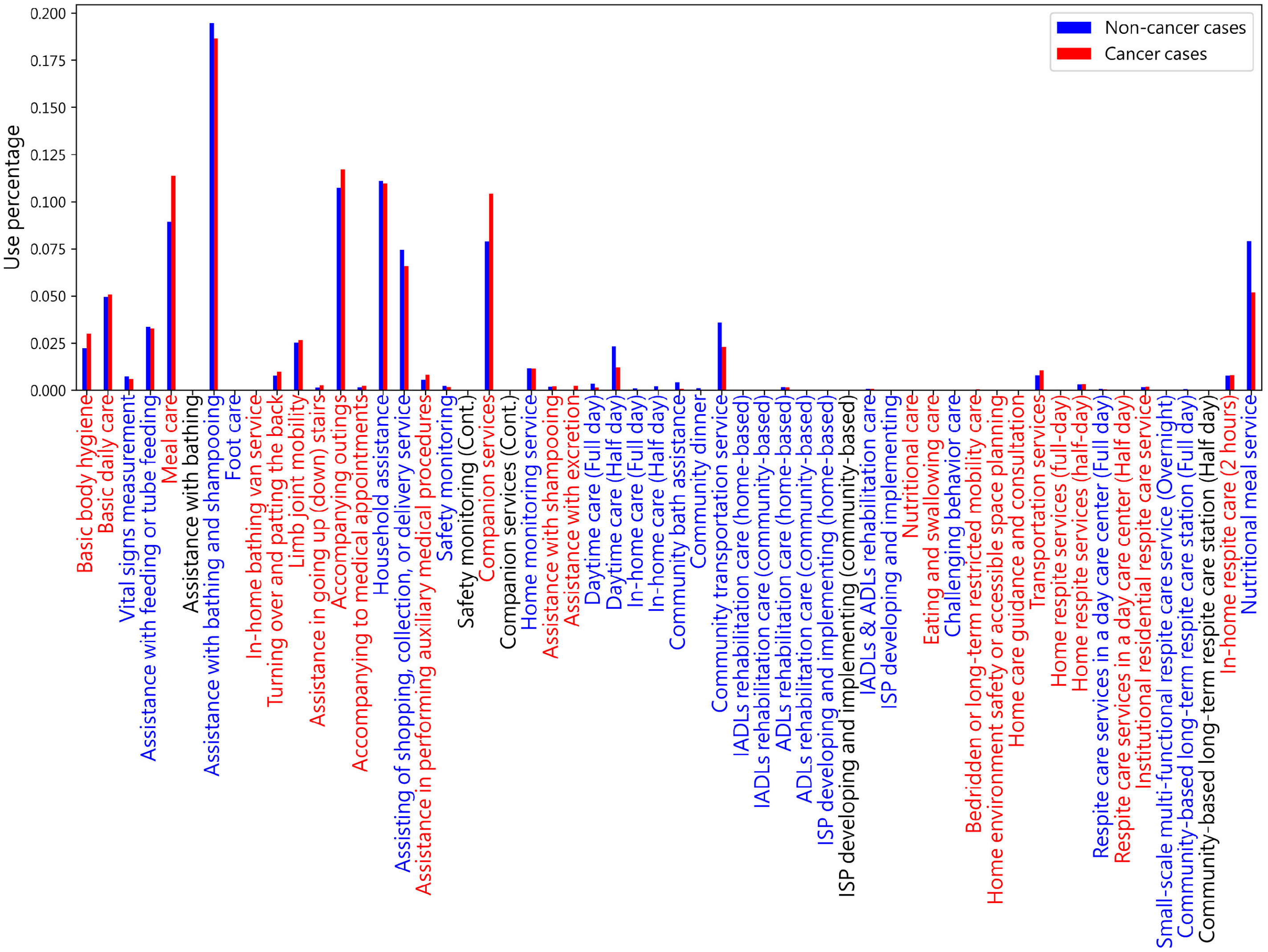

Figure S1. Percentage utilization of LTC services among cancer and non-cancer cases.

In terms of specific LTC services, cancer patients exhibited slightly higher usage rates for basic body hygiene (2.98% vs. 2.24%), basic daily care (5.08% vs. 4.95%), meal care (11.38% vs. 8.92%), and assistance with bathing and shampooing (18.67% vs. 19.48%). Notably, foot care had no reported usage among cancer cases, while it was negligible among non-cancer cases. Accompanying outings (11.72% vs. 10.73%) and companion services (10.42% vs. 7.89%) were more frequently accessed by cancer patients. Conversely, non-cancer patients had higher rates of household assistance (11.10% vs. 10.98%) and assistance with shopping, collection, or delivery services (7.45% vs. 6.58%). Cancer patients showed a higher utilization rate of auxiliary medical procedures (0.82% vs. 0.57%). Finally, non-cancer

cases had a higher utilization rate for nutritional meal services (7.90% vs. 5.18%). These findings provide insights into the differential utilization of LTC services by cancer and non-cancer patients.

We utilized the Mann-Whitney U test to discern the differences in service utilization between cancer and non-cancer cases, which findings are shown in **Table S1**. The data provided includes the number of cases (who used the services at least once), the mean and standard deviation of usage times, and the results of U-statistics. The comparison unveils substantial differences in the usage of various services. In terms of overall service utilization, cancer cases (N = 1,620) exhibited a lower average usage of 203.0 times (SD = 327.9), compared with non-cancer cases (N = 15,481), which had an average usage of 284.4 times (SD = 427.8), signifying a significant difference ($p < 0.001$).

Table S1. Statistical comparison between cases of non-/cancer with LTC service utilization.

Service name	Category	N of cases	N of usage		U- statistics	P-value
			Total	Mean (SD)		
Overall	CA	1,620	328,902	203.0 (327.9)	10,950,163 ***	4.18E-17
	N-CA	15,481	4,402,921	284.4 (427.8)		
Basic personal hygiene	CA	256	10,058	39.3 (75.5)	177,951 ***	5.51E-07
	N-CA	1,724	100,565	58.3 (96.7)		
Basic daily care	CA	271	16,815	62.0 (95.9)	254,289 ***	9.55E-05
	N-CA	2,195	218,402	99.5 (144.0)		
Vital signs measurement	CA	15	1,981	132.1 (153.9)	2,566	0.483913
	N-CA	309	32,310	104.6 (118.7)		
Assistance with feeding or tube feeding	CA	145	10,803	74.5 (104.1)	62,287 ***	0.000252
	N-CA	1,057	148,162	140.2 (206.8)		
Meal care	CA	318	37,279	117.2 (144.3)	434,483 *	0.039369
	N-CA	2,939	392,222	133.5 (150.2)		
Assistance with bathing	CA	-	-	-		
	N-CA	-	-	-		
Assistance with bathing and shampooing	CA	715	61,451	85.9 (109.8)	1,946,622 ***	1.02E-14
	N-CA	6,607	858,287	129.9 (144.1)		
Foot care	CA	-	-	-		
	N-CA	2	2	1.0 (-)		
In-home bathing van service	CA	10	73	7.3 (8.6)	451	0.644594
	N-CA	99	835	8.4 (13.6)		
Turning over and patting the back	CA	48	3,297	68.7 (101.0)	7,163	0.144864
	N-CA	343	34,075	99.3 (136.0)		
Limb Joint Mobility	CA	129	8,793	68.2 (92.6)	63,820 ***	0.003341
	N-CA	1,174	111,642	95.1 (117.4)		
Assistance in going up (down) stairs	CA	8	837	104.6 (120.7)	327	0.448483
	N-CA	70	6,001	85.7 (104.5)		
Accompanying outings	CA	432	38,475	89.1 (170.6)	748,633 ***	9.79E-05
	N-CA	3,912	472,011	120.7 (196.5)		
Accompanying to medical appointments	CA	131	751	5.7 (9.2)	67,267	0.162116
	N-CA	1,108	6,576	5.9 (8.3)		
Household assistance	CA	643	35,928	55.9 (65.7)	1,841,392 ***	2.80E-08
	N-CA	6,602	487,663	73.9 (81.9)		
Assisting of shopping, collection, or delivery service	CA	250	21,637	86.5 (139.4)	340,935	0.061864
	N-CA	2,936	328,156	111.8 (168.7)		
Assistance in performing auxiliary medical procedures	CA	65	2,734	42.1 (65.7)	16,408	0.224519
	N-CA	556	25,117	45.2 (63.3)		
Safety monitoring	CA	3	540	180.0 (115.5)	95	0.452875
	N-CA	50	9,771	195.4 (245.3)		
Safety monitoring (Cont.)	CA	-	-	-		
	N-CA	-	-	-		
Companion services	CA	340	34,154	100.5 (167.1)	444,183 *	0.019534
	N-CA	2,832	346,744	122.4 (189.6)		
Companion services (Cont.)	CA	-	-	-		
	N-CA	-	-	-		
Home monitoring service	CA	21	3,750	178.6 (239.1)	2,177	0.664161
	N-CA	220	50,900	231.4 (298.6)		
Assistance with shampooing	CA	12	673	56.1 (63.5)	920	0.714316

	N-CA	144	7,728	53.7 (69.6)		
Assistance with excretion	CA	12	773	64.4 (90.4)	95	0.368909
	N-CA	13	471	36.2 (69.9)		
Daytime care (full-day)	CA	21	453	21.6 (40.7)	3,188	0.354288
	N-CA	345	14,797	42.9 (76.9)		
Daytime care (half-day)	CA	56	3,915	69.9 (96.9)	26,791	0.102008
	N-CA	1,099	102,440	93.2 (112.5)		
In-home care (full-day)	CA	2	11	5.5 (3.5)	31	0.364135
	N-CA	50	3,932	78.6 (122.6)		
In-home care (half-day)	CA	1	15	15.0 (-)	24	0.381526
	N-CA	98	8,775	89.5 (118.6)		
Community bath assistance	CA	12	210	17.5 (26.6)	1,100 *	0.03194
	N-CA	289	18,010	62.3 (90.6)		
Community dinner	CA	1	15	15.0 (-)	9	0.374114
	N-CA	38	4,467	117.6 (158.9)		
Community transportation service	CA	59	7,504	127.2 (179.8)	32,169	0.350509
	N-CA	1,175	156,737	133.4 (180.3)		
IADLs rehabilitation care (home-based)	CA	1	1	1.0 (-)	5	0.240184
	N-CA	32	199	6.2 (4.8)		
IADLs rehabilitation care (community-based)	CA	-	-	-		
	N-CA	3	23	7.7 (2.3)		
ADLs rehabilitation care (home-based)	CA	79	535	6.8 (6.2)	30,929 *	0.019029
	N-CA	929	7,295	7.9 (5.7)		
ADLs rehabilitation care (community-based)	CA	-	-	-		
	N-CA	4	36	9.0 (7.5)		
ISP developing and implementing (home-based)	CA	-	-	-		
	N-CA	9	70	7.8 (4.6)		
ISP developing and implementing (community-based)	CA	-	-	-		
	N-CA	-	-	-		
IADLs & ADLs rehabilitation care	CA	51	223	4.4 (3.3)	16,699	0.594374
	N-CA	685	3,083	4.5 (3.2)		
ISP developing and implementing	CA	-	-	-		
	N-CA	2	10	5.0 (1.4)		
Nutritional care	CA	7	8	1.1 (0.4)	53	0.15681
	N-CA	22	45	2.0 (1.5)		
Eating and swallowing care	CA	10	35	3.5 (3.3)	285	0.213452
	N-CA	75	356	4.7 (3.8)		
Challenging behavior care	CA	-	-	-		
	N-CA	6	14	2.3 (2.0)		
Bedridden or long-term restricted mobility care	CA	34	164	4.8 (4.0)	3,896	0.300203
	N-CA	257	1,509	5.9 (5.0)		
Home environment safety or accessible space planning	CA	10	12	1.2 (0.4)	411	0.992726
	N-CA	82	99	1.2 (0.4)		
Home care guidance and consultation	CA	16	47	2.9 (2.7)	481	0.124569
	N-CA	79	318	4.0 (4.0)		
Transportation services	CA	279	3,506	12.6 (18.4)	307,850	0.774921
	N-CA	2,230	35,004	15.7 (26.3)		
Home respite services (full-day)	CA	29	102	3.5 (3.5)	4,326	0.610933
	N-CA	316	1,208	3.8 (3.7)		
Home respite services (half-day)	CA	109	1,024	9.4 (9.9)	53,950	0.086254
	N-CA	1,099	13,067	11.9 (12.1)		
Respite care services in a day care center (full-day)	CA	10	135	13.5 (16.6)	1,846	0.30255
	N-CA	310	3,016	9.7 (11.3)		
Respite care services in a day care center (half-day)	CA	5	33	6.6 (6.9)	130	0.768335
	N-CA	48	302	6.3 (9.1)		
Institutional residential respite care service	CA	45	582	12.9 (5.9)	12,102	0.934877
	N-CA	534	6,899	12.9 (6.8)		
Small-scale multi-functional respite care service (overnight)	CA	-	-	-		
	N-CA	9	48	5.3 (3.7)		
Community-based long-term respite care station (full day)	CA	-	-	-		
	N-CA	15	2,706	180.4 (175.2)		
Community-based long-term respite care station (half day)	CA	-	-	-		
	N-CA	-	-	-		
In-home respite care	CA	213	2,649	12.4 (13.6)	218,269 ***	0.004354

(2 hours)	N-CA	2,323	34,053	14.7 (13.7)		
Nutritional meal service	CA	77	16,921	219.8 (207.3)	34,797 ***	0.004715
	N-CA	1,119	346,763	309.9 (279.6)		

Abbreviation: N = Number; SD = Standard deviation; CA = Cancer; N-CA = Non-cancer; CONT = continue; ADL = Activities of Daily Living; IADL = Instrumental Activities of Daily Living; ISP = Individualized Support Plan
Noted: * = P < 0.05; ** = P < 0.01; *** = P < 0.001

In terms of specific services, the usage of basic personal hygiene was notably lower in cancer patients (39.3, SD = 75.5) compared to non-cancer patients (58.3, SD = 96.7, p < 0.001). Significant differences were also observed in services such as basic daily care (p < 0.001), assistance with feeding or tube feeding (p = 0.000252), meal care (p = 0.039369), assistance with bathing and shampooing (p < 0.001), limb joint mobility (p = 0.003341), accompaniment on outings (p < 0.001), household assistance (p < 0.001), ADLs rehabilitation care at home (p = 0.019029), companion services (p = 0.019534), community bath assistance (p = 0.03194), in-home respite care (2 hours, p = 0.004354), and nutritional meal service (p = 0.004715).

Conversely, no significant differences were observed between the two groups for a range of services such as vital signs measurement, assistance with ascending and descending stairs, in-home care, rehabilitation care, nutritional care, and respite care services, among others. Additionally, some specific services, including continuous safety monitoring, home-based ADLs rehabilitation care, challenging behavior care, and community-based long-term respite care station, were not utilized by any of the cancer cases in this sample.

Supplementary Figures

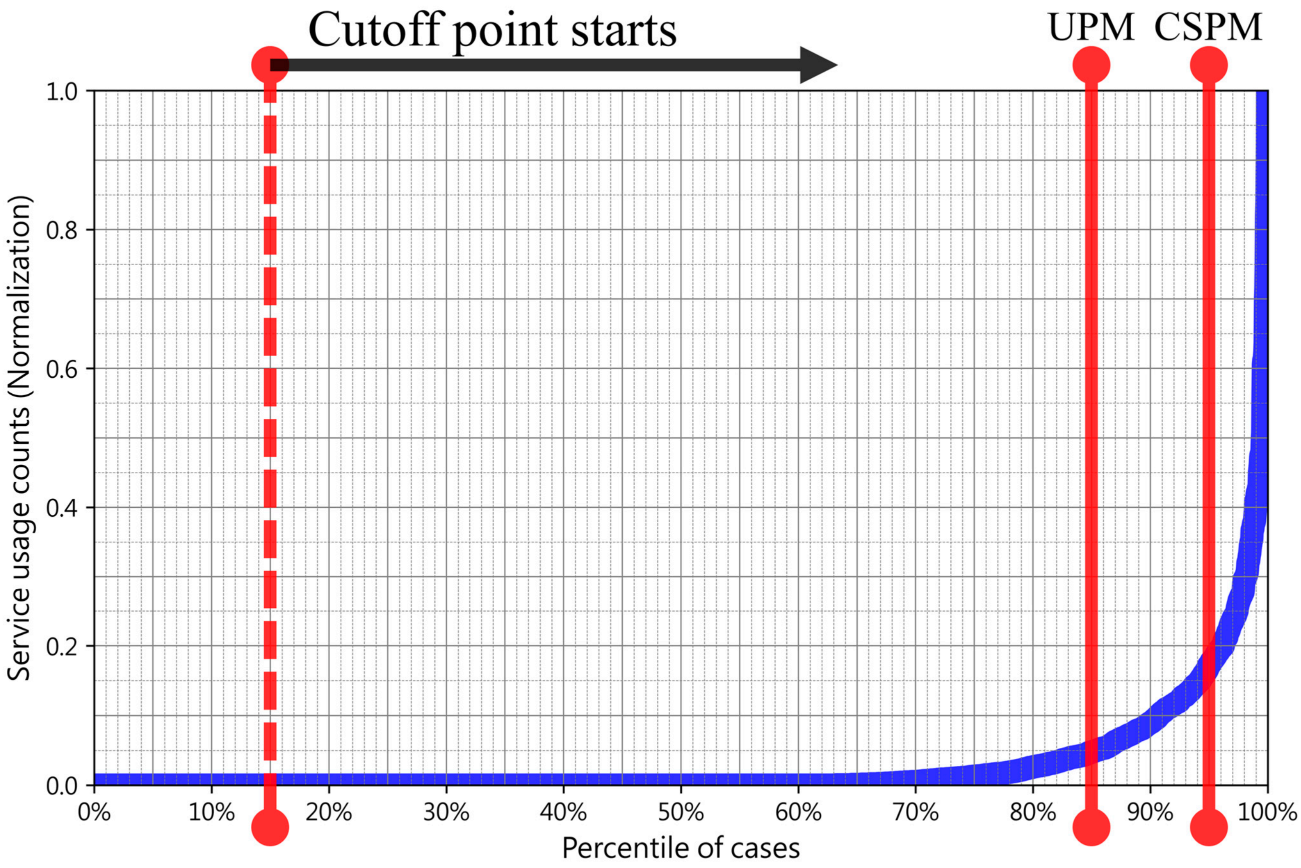

Figure S2. Conceptualization of sensitivity analysis. We sorted all cancer cases in ascending order based on their total usage times of services. The "percentile" shows where a case ranks in terms of service usage compared to others. For the Unified Prediction Model (UPM), we treat all service categories equally by setting the upper cutoff point at 85%. In contrast, for the Category-Specific Prediction Models (CSPMs), we created specific prediction models for the top five most frequently used service categories and set the upper cutoff point at 95%.

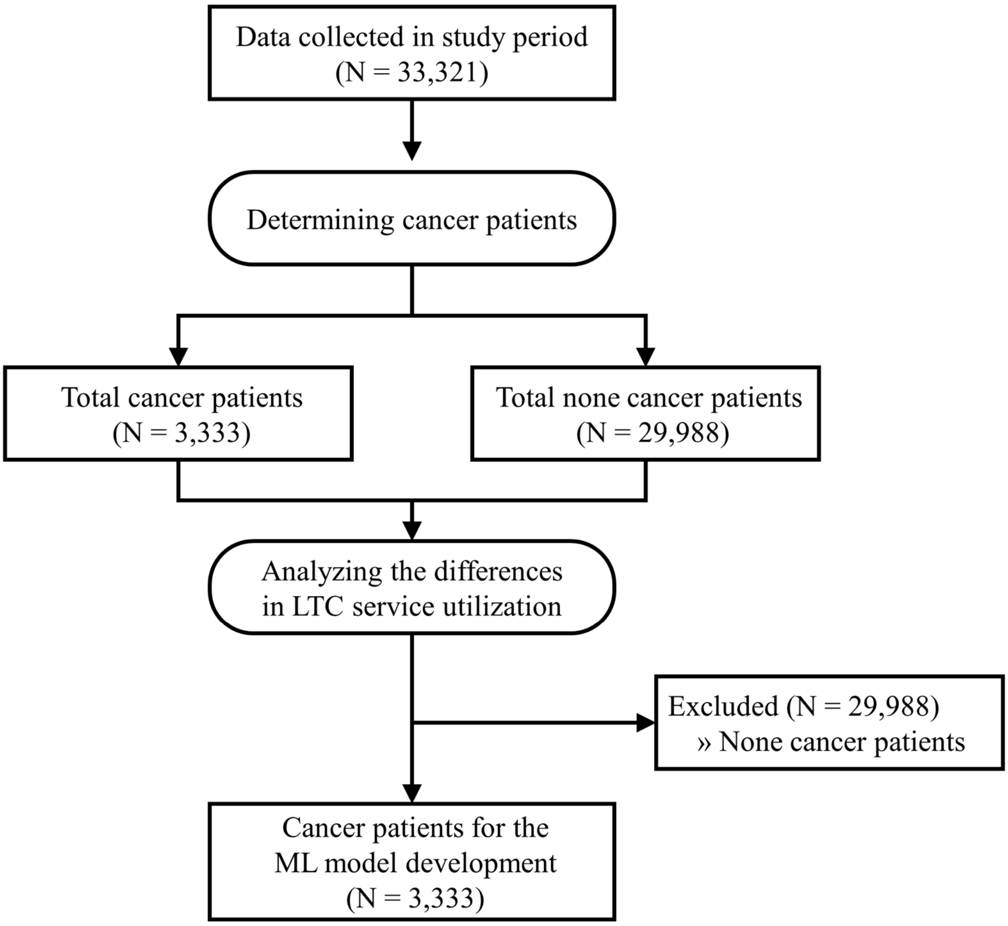

Figure S3. Cohort selection process in our study.

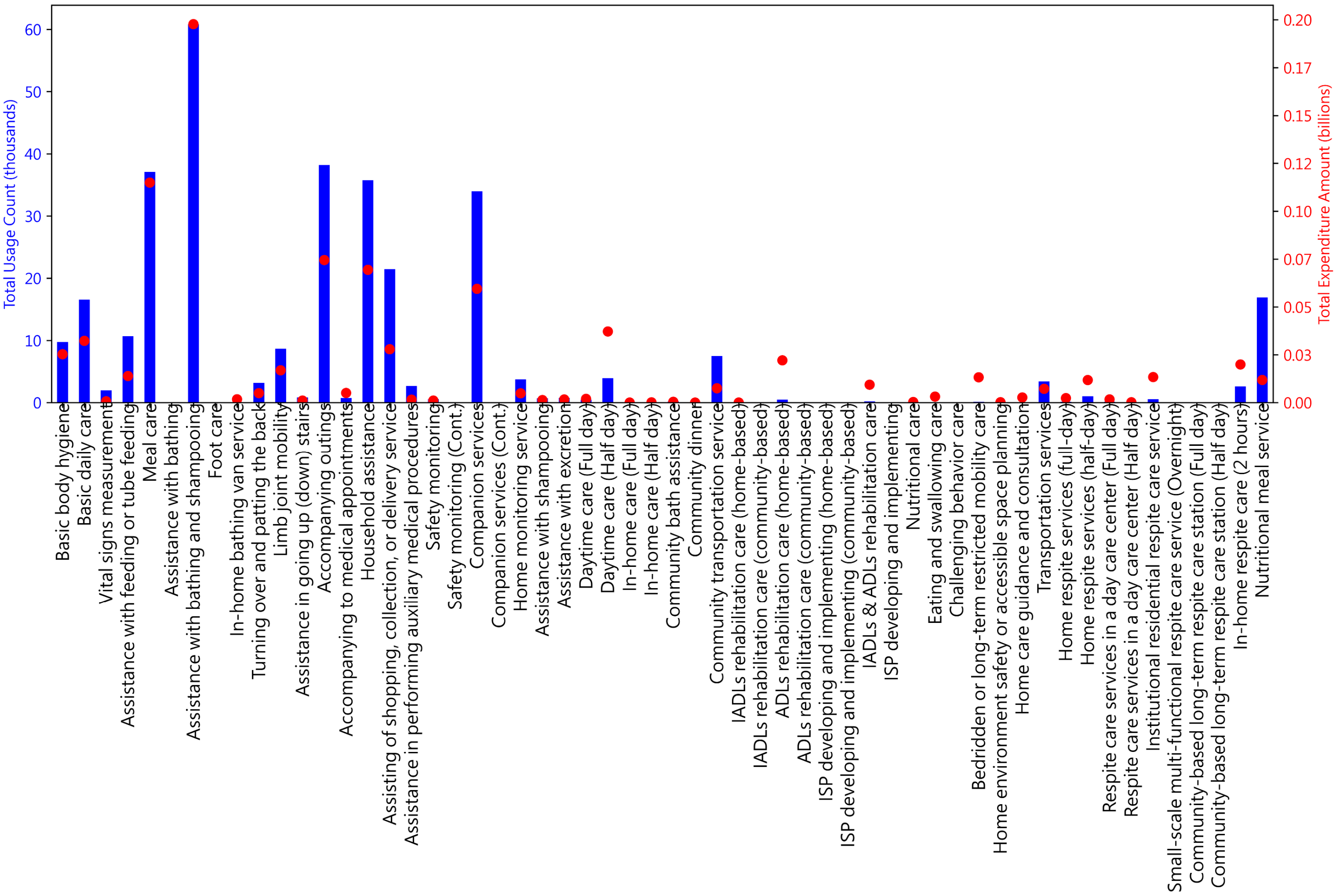

Figure S4. Numbers of usages and costs for LTC services among cancer cases.

Supplementary Tables

Table S2. Summary of service categories provided by Taiwanese LTC 2.0.

**Service categories**	
**Homecare, daycare and adult foster service**	**Professional service**
Basic personal hygiene	IADLs rehabilitation care (home-based)
Basic daily care	IADLs rehabilitation care (community-based)
Vital signs measurement	ADLs rehabilitation care (home-based)
Assistance with feeding or tube	ADLs rehabilitation care (community-based)
feeding	ISP developing and implementing (home-based)
Meal care	ISP developing and implementing (community-based)
Assistance with bathing	IADLs & ADLs rehabilitation care
Assistance with bathing and shampooing	ISP developing and implementing
Foot care	Nutritional care
In-home bathing van service	Eating and swallowing care
Turning over and patting the back	Challenging behavior care
Limb Joint Mobility	Bedridden or long-term restricted mobility care
Assistance in going up (down) stairs	Home environment safety or accessible space planning
Accompanying outings	Home care guidance and consultation
Accompanying to medical appointments	**Transportation**
Household assistance	Transportation services
Assisting of shopping, collection, or delivery service	**Respite service**
Assistance in performing auxiliary medical procedures	Home respite services (full-day)
Safety monitoring	Home respite services (half-day)
Safety monitoring (Cont.)	Respite care services in a daycare center (full-day)
Companion services	Respite care services in a daycare center (half-day)
Companion services (Cont.)	Institutional residential respite care service
Home monitoring service	Small-scale multi-functional respite care service (overnight)
Assistance with shampooing	Community-based long-term respite care station (full-day)
Assistance with excretion	Community-based long-term respite care station (half-day)
Daytime care (full-day)	In-home respite care (2 hours)
Daytime care (half-day)	**Others**
In-home care (full-day)	Nutritional meal service
In-home care (half-day)	
Community bath assistance	
Community dinner	
Community transportation service	

Abbreviation: ADL = activities of daily living; IADL = instrumental activities of daily living

Table S3. Features used in this study.

Feature Name	Total	NoF	Type
**Related to case (care recipient)**	406		
Case basic information		28	C & N
Case evaluation complete weekday and month		19	C
Case disability type		12	C & N
Case communication status		6	C & N
Case short-term memory ability		6	C & N
Case ADL and IADL status		22	C & N
Case pain, wound, and joint condition		30	C
Case nutrition and frailty condition		9	C & N
Case exists illness status		70	C
Case exists cancer status		26	C
Case medical assistance demand status		26	C
Case dysphagia status		7	C
Case training history status		5	C
Case fall history status		8	C
Case cohabitation status		18	C
Case living environment status		21	C
Case social connection status		7	C
Case BPSD status		64	C
Case caregiving status		9	C & N
Case and Caregiver relationship		13	C
**Related with caregiver**	56		
Caregiver basic information		3	C & N
Caregiver burden status		17	C & N
Caregiver living and working status		36	C & N

Total Features: 462

Abbreviations: NoF = Number of Features; C = Categorical; N = Numerical; ADL = Activities of daily living; IADL = Instrumental Activities of Daily Living; BPSD = Behavioral and psychological symptoms of dementia

Table S4. Demographics of the datasets.

Characteristic	Entire (N = 33,321)	Cancer patients (N = 3,333)	Non-cancer patients (N = 29,988)
**Age, mean (SD), years**			
Case	75.6 (12.6)	74.88 (10.1)	76.0 (12.8)
Caregiver	51.7 (20.9)	53.16 (19.4)	51.5 (21.1)
**Male, N (%)**			
Case	14,267 (42.8)	1,777 (53.3)	12,490 (41.6)
Caregiver	12,237 (36.7)	1,146 (34.3)	11,091 (37.0)
**Hiring an employed caregiver**	3,786 (11.4)	283 (8.5)	3,503 (11.7)

Abbreviations: N = Number of cases; SD = Standard deviation

Table S5. Results of sensitivity analysis (performance measured by AUROC).

COP	COV	RFs	NoLs		LASSO																LASSO+SMOTE															
			0	1	AB	BC	DT	ET	GB	GNB	GP	KNN	LDA	LR	MLP	MNB	QDA	RF	SVM	XGB	AB	BC	DT	ET	GB	GNB	GP	KNN	LDA	LR	MLP	MNB	QDA	RF	SVM	XGB
54	1.28	1	193	141	0.53	0.53	0.53	0.53	0.53	0.53	0.53	0.53	0.53	0.53	0.53	0.50	0.53	0.53	0.53	0.53	0.53	0.53	0.53	0.53	0.53	0.53	0.53	0.53	0.53	0.53	0.53	0.50	0.53	0.53	0.53	0.53
54.5	1.28	1	193	141	0.53	0.53	0.53	0.53	0.53	0.53	0.53	0.53	0.53	0.53	0.53	0.50	0.53	0.53	0.53	0.53	0.53	0.53	0.53	0.53	0.53	0.53	0.53	0.53	0.53	0.53	0.53	0.50	0.53	0.53	0.53	0.53
55	1.28	1	193	141	0.53	0.53	0.53	0.53	0.53	0.53	0.53	0.53	0.53	0.53	0.53	0.50	0.53	0.53	0.53	0.53	0.53	0.53	0.53	0.53	0.53	0.53	0.53	0.53	0.53	0.53	0.53	0.50	0.53	0.53	0.53	0.53
55.5	1.28	1	193	141	0.53	0.53	0.53	0.53	0.53	0.53	0.53	0.53	0.53	0.53	0.53	0.50	0.53	0.53	0.53	0.53	0.53	0.53	0.53	0.53	0.53	0.53	0.53	0.53	0.53	0.53	0.53	0.50	0.53	0.53	0.53	0.53
56	1.28	1	193	141	0.53	0.53	0.53	0.53	0.53	0.53	0.53	0.53	0.53	0.53	0.53	0.50	0.53	0.53	0.53	0.53	0.53	0.53	0.53	0.53	0.53	0.53	0.53	0.53	0.53	0.53	0.53	0.50	0.53	0.53	0.53	0.53
56.5	3	57	199	135	0.63	0.62	0.54	0.64	0.68	0.59	0.62	0.54	0.63	0.66	0.62	0.64	0.66	0.62	0.57	0.66	0.65	0.63	0.56	0.68	0.68	0.62	0.61	0.60	0.66	0.66	0.67	0.62	0.68	0.67	0.67	0.68
57	3.24	57	202	132	0.63	0.63	0.52	0.65	0.67	0.61	0.63	0.55	0.63	0.68	0.65	0.62	0.68	0.66	0.55	0.69	0.67	0.69	0.55	0.67	0.67	0.64	0.62	0.66	0.68	0.67	0.68	0.63	0.69	0.67	0.67	0.69
57.5	3.24	63	202	132	0.63	0.63	0.52	0.65	0.67	0.61	0.63	0.55	0.63	0.68	0.65	0.62	0.68	0.66	0.55	0.69	0.67	0.69	0.55	0.67	0.67	0.64	0.62	0.66	0.68	0.67	0.68	0.63	0.69	0.67	0.67	0.69
58	3.24	63	202	132	0.63	0.63	0.52	0.65	0.67	0.61	0.63	0.55	0.63	0.68	0.65	0.62	0.68	0.66	0.55	0.69	0.67	0.69	0.55	0.67	0.67	0.64	0.62	0.66	0.68	0.67	0.68	0.63	0.69	0.67	0.67	0.69
58.5	5	63	209	125	0.64	0.59	0.55	0.65	0.66	0.61	0.61	0.53	0.65	0.67	0.66	0.63	0.66	0.68	0.55	0.67	0.67	0.67	0.54	0.68	0.67	0.65	0.59	0.65	0.68	0.67	0.66	0.63	0.67	0.70	0.68	0.68
59	5.88	63	212	122	0.64	0.64	0.56	0.66	0.68	0.60	0.61	0.55	0.64	0.69	0.67	0.62	0.65	0.65	0.55	0.69	0.68	0.65	0.56	0.70	0.68	0.66	0.61	0.62	0.67	0.68	0.67	0.64	0.68	0.71	0.68	0.67
59.5	5.88	57	212	122	0.64	0.64	0.56	0.66	0.68	0.60	0.61	0.55	0.64	0.69	0.67	0.62	0.65	0.65	0.55	0.69	0.68	0.65	0.56	0.70	0.68	0.66	0.61	0.62	0.67	0.68	0.67	0.64	0.68	0.71	0.68	0.67
60	7	57	213	121	0.62	0.66	0.53	0.64	0.66	0.59	0.60	0.50	0.63	0.66	0.65	0.61	0.63	0.66	0.55	0.67	0.66	0.66	0.56	0.67	0.67	0.64	0.59	0.62	0.67	0.67	0.66	0.63	0.68	0.67	0.66	0.67
60.5	8	62	214	120	0.63	0.64	0.52	0.66	0.66	0.59	0.59	0.57	0.63	0.66	0.64	0.62	0.65	0.67	0.61	0.68	0.66	0.63	0.57	0.68	0.69	0.63	0.59	0.60	0.67	0.67	0.66	0.61	0.68	0.68	0.66	0.66
61	8	67	214	120	0.63	0.64	0.52	0.66	0.66	0.59	0.59	0.57	0.63	0.66	0.64	0.62	0.65	0.67	0.61	0.68	0.66	0.63	0.57	0.68	0.69	0.63	0.59	0.60	0.67	0.67	0.66	0.61	0.68	0.68	0.66	0.66
61.5	9	61	215	119	0.59	0.61	0.57	0.64	0.64	0.55	0.58	0.55	0.60	0.67	0.64	0.57	0.62	0.64	0.55	0.64	0.65	0.67	0.57	0.67	0.67	0.61	0.60	0.63	0.66	0.66	0.66	0.60	0.67	0.65	0.67	0.68
62	10	61	216	118	0.59	0.59	0.51	0.61	0.63	0.55	0.59	0.52	0.62	0.66	0.60	0.58	0.60	0.58	0.53	0.62	0.65	0.64	0.58	0.65	0.65	0.61	0.58	0.62	0.65	0.66	0.65	0.58	0.64	0.65	0.66	0.66
62.5	11	55	217	117	0.64	0.65	0.58	0.62	0.64	0.58	0.59	0.56	0.63	0.67	0.67	0.59	0.63	0.64	0.57	0.67	0.66	0.66	0.61	0.68	0.65	0.62	0.60	0.63	0.66	0.66	0.67	0.60	0.66	0.67	0.66	0.69
63	12	55	218	116	0.60	0.60	0.53	0.62	0.63	0.55	0.57	0.53	0.63	0.67	0.59	0.57	0.61	0.62	0.55	0.66	0.65	0.64	0.56	0.63	0.65	0.60	0.59	0.63	0.66	0.66	0.65	0.58	0.65	0.66	0.64	0.66
63.5	13	63	221	113	0.60	0.65	0.53	0.63	0.66	0.56	0.60	0.61	0.61	0.65	0.63	0.57	0.62	0.65	0.58	0.65	0.65	0.66	0.58	0.66	0.65	0.60	0.61	0.61	0.66	0.66	0.66	0.60	0.66	0.66	0.63	0.65
64	14	60	222	112	0.60	0.62	0.52	0.63	0.64	0.55	0.59	0.56	0.60	0.65	0.64	0.56	0.61	0.63	0.60	0.64	0.65	0.63	0.61	0.68	0.67	0.61	0.59	0.56	0.66	0.66	0.65	0.59	0.66	0.65	0.65	0.65
64.5	14	56	222	112	0.60	0.62	0.52	0.63	0.64	0.55	0.59	0.56	0.60	0.65	0.64	0.56	0.61	0.63	0.60	0.64	0.65	0.63	0.61	0.68	0.67	0.61	0.59	0.56	0.66	0.66	0.65	0.59	0.66	0.65	0.65	0.65
65	16	53	226	108	0.60	0.62	0.60	0.66	0.61	0.56	0.59	0.57	0.60	0.67	0.61	0.58	0.61	0.64	0.62	0.64	0.66	0.65	0.58	0.66	0.70	0.61	0.59	0.60	0.66	0.66	0.66	0.60	0.66	0.65	0.65	0.66
65.5	18	56	229	105	0.61	0.65	0.51	0.65	0.63	0.57	0.58	0.60	0.60	0.66	0.58	0.56	0.60	0.62	0.60	0.66	0.65	0.64	0.50	0.64	0.68	0.62	0.62	0.61	0.65	0.66	0.66	0.60	0.65	0.67	0.67	0.65
66	19	57	230	104	0.61	0.61	0.54	0.60	0.64	0.56	0.54	0.59	0.60	0.67	0.65	0.57	0.60	0.61	0.54	0.63	0.67	0.64	0.56	0.65	0.65	0.61	0.60	0.60	0.66	0.66	0.67	0.60	0.66	0.67	0.67	0.66
66.5	20.78	56	232	102	0.61	0.58	0.57	0.61	0.66	0.57	0.57	0.60	0.61	0.66	0.66	0.56	0.61	0.63	0.59	0.67	0.66	0.64	0.52	0.66	0.67	0.63	0.60	0.66	0.66	0.66	0.66	0.60	0.67	0.67	0.66	0.71
67	22	56	233	101	0.61	0.62	0.54	0.64	0.63	0.57	0.59	0.58	0.60	0.66	0.64	0.56	0.60	0.64	0.62	0.70	0.66	0.65	0.59	0.66	0.68	0.63	0.62	0.67	0.66	0.67	0.65	0.59	0.67	0.67	0.63	0.68
67.5	23	34	235	99	0.61	0.59	0.52	0.61	0.67	0.56	0.56	0.53	0.60	0.65	0.64	0.57	0.61	0.61	0.59	0.64	0.66	0.63	0.59	0.66	0.67	0.63	0.59	0.62	0.65	0.67	0.67	0.60	0.67	0.67	0.64	0.70
68	24	37	236	98	0.60	0.64	0.53	0.65	0.65	0.57	0.56	0.62	0.61	0.69	0.66	0.57	0.62	0.65	0.60	0.67	0.70	0.68	0.61	0.67	0.70	0.66	0.63	0.63	0.68	0.69	0.69	0.64	0.71	0.67	0.67	0.70
68.5	26	68	239	95	0.59	0.64	0.53	0.64	0.61	0.55	0.56	0.60	0.59	0.67	0.67	0.54	0.61	0.63	0.58	0.67	0.68	0.66	0.55	0.67	0.69	0.64	0.63	0.64	0.68	0.67	0.66	0.62	0.68	0.69	0.65	0.69

69	27.08	39	241	93	0.61	0.64	0.57	0.62	0.65	0.56	0.57	0.60	0.60	0.67	0.66	0.56	0.61	0.63	0.57	0.68	0.67	0.65	0.51	0.67	0.69	0.66	0.62	0.60	0.67	0.69	0.67	0.63	0.70	0.68	0.67	0.68
69.5	29	36	243	91	0.61	0.65	0.51	0.63	0.63	0.57	0.56	0.60	0.60	0.69	0.68	0.58	0.62	0.63	0.57	0.69	0.69	0.69	0.61	0.67	0.68	0.65	0.62	0.65	0.68	0.68	0.68	0.63	0.71	0.69	0.65	0.70
70	31	36	246	88	0.58	0.61	0.49	0.60	0.62	0.55	0.55	0.58	0.58	0.68	0.63	0.55	0.60	0.61	0.54	0.63	0.68	0.68	0.56	0.67	0.67	0.65	0.61	0.62	0.68	0.68	0.66	0.64	0.69	0.66	0.68	0.69
70.5	32	37	249	85	0.58	0.60	0.54	0.62	0.63	0.55	0.53	0.55	0.56	0.69	0.66	0.57	0.59	0.62	0.54	0.65	0.69	0.68	0.53	0.70	0.70	0.67	0.64	0.61	0.68	0.68	0.67	0.65	0.71	0.67	0.69	0.71
71	34	60	253	81	0.59	0.66	0.56	0.63	0.63	0.56	0.57	0.61	0.58	0.67	0.61	0.56	0.61	0.64	0.60	0.65	0.67	0.66	0.63	0.71	0.68	0.65	0.63	0.67	0.67	0.68	0.66	0.64	0.70	0.69	0.65	0.65
71.5	36	59	254	80	0.57	0.61	0.53	0.62	0.57	0.55	0.56	0.61	0.57	0.68	0.60	0.57	0.59	0.63	0.58	0.65	0.69	0.68	0.57	0.68	0.68	0.65	0.63	0.59	0.68	0.67	0.68	0.63	0.71	0.68	0.66	0.65
72	37.04	39	256	78	0.60	0.62	0.53	0.65	0.60	0.58	0.59	0.59	0.58	0.68	0.64	0.56	0.61	0.64	0.60	0.67	0.69	0.69	0.57	0.69	0.66	0.65	0.62	0.68	0.69	0.69	0.68	0.62	0.72	0.71	0.68	0.71
72.5	40	38	259	75	0.58	0.62	0.56	0.62	0.61	0.55	0.54	0.54	0.58	0.69	0.61	0.56	0.59	0.64	0.57	0.64	0.71	0.71	0.63	0.71	0.66	0.66	0.62	0.65	0.69	0.69	0.66	0.63	0.70	0.68	0.68	0.68
73	42	44	259	75	0.59	0.61	0.55	0.62	0.63	0.57	0.56	0.60	0.59	0.70	0.60	0.58	0.59	0.64	0.58	0.66	0.68	0.65	0.55	0.68	0.67	0.64	0.63	0.62	0.70	0.70	0.68	0.63	0.70	0.69	0.67	0.68
73.5	45	38	260	74	0.59	0.55	0.57	0.59	0.60	0.55	0.57	0.56	0.58	0.68	0.60	0.57	0.58	0.58	0.58	0.60	0.68	0.66	0.58	0.65	0.68	0.62	0.60	0.57	0.68	0.69	0.68	0.62	0.67	0.68	0.65	0.62
74	50.36	37	261	73	0.60	0.58	0.54	0.62	0.59	0.55	0.56	0.55	0.58	0.67	0.62	0.56	0.58	0.59	0.56	0.60	0.68	0.67	0.56	0.65	0.64	0.63	0.60	0.59	0.68	0.69	0.67	0.61	0.69	0.67	0.66	0.65
74.5	53	46	262	72	0.59	0.59	0.48	0.61	0.60	0.56	0.56	0.54	0.57	0.66	0.58	0.56	0.57	0.59	0.55	0.64	0.68	0.66	0.62	0.68	0.68	0.64	0.61	0.61	0.67	0.67	0.64	0.62	0.68	0.67	0.66	0.66
75	55	44	263	71	0.58	0.61	0.54	0.64	0.61	0.58	0.55	0.52	0.58	0.69	0.59	0.57	0.60	0.62	0.56	0.65	0.70	0.68	0.65	0.67	0.70	0.64	0.61	0.60	0.68	0.69	0.67	0.62	0.69	0.70	0.66	0.67
75.5	59	35	265	69	0.56	0.61	0.49	0.59	0.59	0.55	0.58	0.59	0.55	0.68	0.61	0.54	0.58	0.58	0.59	0.59	0.68	0.68	0.53	0.69	0.69	0.63	0.63	0.59	0.67	0.67	0.68	0.62	0.68	0.68	0.63	0.65
76	63	38	266	68	0.59	0.59	0.53	0.59	0.58	0.57	0.60	0.58	0.59	0.69	0.58	0.57	0.59	0.59	0.59	0.61	0.69	0.67	0.56	0.67	0.68	0.64	0.63	0.60	0.68	0.70	0.68	0.64	0.67	0.67	0.64	0.62
76.5	66	42	267	67	0.58	0.61	0.55	0.62	0.59	0.57	0.61	0.54	0.58	0.68	0.58	0.57	0.60	0.61	0.61	0.61	0.68	0.65	0.55	0.65	0.70	0.64	0.64	0.63	0.68	0.68	0.68	0.64	0.69	0.68	0.61	0.66
77	70	39	268	66	0.58	0.61	0.59	0.58	0.57	0.59	0.58	0.52	0.57	0.68	0.57	0.57	0.58	0.58	0.59	0.61	0.67	0.64	0.55	0.65	0.67	0.65	0.60	0.66	0.67	0.67	0.68	0.63	0.68	0.67	0.63	0.65
77.5	74	42	270	64	0.60	0.59	0.53	0.58	0.58	0.59	0.57	0.59	0.58	0.68	0.64	0.57	0.60	0.61	0.54	0.61	0.68	0.65	0.63	0.68	0.67	0.66	0.59	0.66	0.67	0.67	0.68	0.63	0.69	0.67	0.65	0.63
78	80	38	271	63	0.59	0.60	0.58	0.60	0.59	0.58	0.59	0.59	0.58	0.67	0.59	0.57	0.59	0.58	0.56	0.60	0.69	0.71	0.58	0.66	0.67	0.65	0.61	0.61	0.68	0.69	0.67	0.65	0.67	0.65	0.60	0.64
78.5	86	38	271	63	0.58	0.59	0.54	0.61	0.57	0.58	0.60	0.57	0.56	0.68	0.56	0.57	0.60	0.60	0.56	0.58	0.66	0.64	0.55	0.66	0.65	0.65	0.61	0.64	0.68	0.68	0.67	0.65	0.67	0.66	0.61	0.63
79	90.28	43	273	61	0.58	0.59	0.57	0.60	0.57	0.58	0.57	0.55	0.58	0.68	0.61	0.58	0.60	0.58	0.55	0.61	0.68	0.64	0.58	0.63	0.66	0.66	0.60	0.64	0.69	0.69	0.67	0.67	0.71	0.64	0.60	0.65
79.5	94	43	275	59	0.57	0.58	0.51	0.57	0.53	0.57	0.58	0.54	0.55	0.66	0.57	0.57	0.57	0.60	0.57	0.59	0.67	0.65	0.56	0.65	0.64	0.66	0.61	0.64	0.67	0.68	0.67	0.65	0.67	0.66	0.60	0.61
80	101.6	34	275	59	0.56	0.61	0.58	0.61	0.59	0.58	0.60	0.58	0.56	0.68	0.57	0.56	0.58	0.61	0.59	0.60	0.67	0.66	0.55	0.65	0.66	0.65	0.64	0.66	0.67	0.68	0.65	0.65	0.68	0.68	0.54	0.62
80.5	107	31	277	57	0.57	0.60	0.58	0.60	0.58	0.58	0.62	0.54	0.57	0.66	0.59	0.54	0.57	0.57	0.58	0.60	0.66	0.59	0.57	0.61	0.65	0.64	0.64	0.66	0.67	0.66	0.65	0.62	0.65	0.62	0.55	0.62
81	111.92	42	278	56	0.58	0.61	0.61	0.61	0.62	0.59	0.61	0.58	0.56	0.66	0.61	0.57	0.59	0.61	0.60	0.61	0.67	0.69	0.53	0.65	0.66	0.66	0.64	0.66	0.67	0.68	0.64	0.64	0.68	0.66	0.60	0.63
81.5	120.58	42	280	54	0.58	0.59	0.54	0.60	0.59	0.59	0.61	0.57	0.57	0.67	0.58	0.57	0.59	0.60	0.59	0.60	0.67	0.67	0.60	0.65	0.64	0.65	0.63	0.66	0.68	0.68	0.64	0.63	0.68	0.63	0.65	0.63
82	131.24	41	281	53	0.56	0.59	0.55	0.59	0.57	0.59	0.59	0.55	0.56	0.68	0.61	0.57	0.58	0.58	0.58	0.57	0.69	0.63	0.59	0.65	0.68	0.66	0.65	0.65	0.68	0.69	0.66	0.66	0.69	0.65	0.64	0.64
82.5	141	38	281	53	0.56	0.59	0.54	0.60	0.53	0.59	0.57	0.56	0.55	0.68	0.54	0.57	0.58	0.59	0.57	0.54	0.69	0.62	0.58	0.62	0.65	0.68	0.63	0.61	0.70	0.69	0.68	0.68	0.69	0.66	0.57	0.60
83	150	40	282	52	0.57	0.56	0.55	0.62	0.57	0.61	0.58	0.55	0.57	0.68	0.57	0.60	0.60	0.62	0.56	0.57	0.71	0.65	0.57	0.63	0.68	0.68	0.64	0.61	0.69	0.70	0.67	0.68	0.71	0.69	0.66	0.60
83.5	157.22	44	282	52	0.59	0.59	0.55	0.59	0.52	0.60	0.57	0.54	0.57	0.70	0.55	0.60	0.60	0.60	0.56	0.54	0.70	0.66	0.55	0.65	0.66	0.70	0.62	0.56	0.71	0.70	0.66	0.69	0.70	0.67	0.58	0.60
84	167.88	22	284	50	0.58	0.61	0.59	0.62	0.54	0.61	0.59	0.60	0.57	0.71	0.53	0.60	0.59	0.61	0.58	0.58	0.71	0.65	0.55	0.68	0.66	0.70	0.62	0.61	0.72	0.73	0.68	0.70	0.71	0.68	0.61	0.61
84.5	175.54	21	284	50	0.58	0.66	0.61	0.67	0.63	0.60	0.62	0.64	0.59	0.62	0.59	0.57	0.60	0.67	0.66	0.66	0.64	0.66	0.58	0.64	0.64	0.64	0.65	0.59	0.64	0.63	0.63	0.62	0.65	0.63	0.50	0.64
85	185.2	13	286	48	0.59	0.60	0.58	0.59	0.57	0.62	0.59	0.61	0.58	0.61	0.57	0.56	0.60	0.60	0.54	0.57	0.62	0.61	0.53	0.61	0.58	0.64	0.60	0.57	0.63	0.61	0.62	0.58	0.63	0.62	0.51	0.60

Abbreviation: COP = Cutoff Percentile; COV = Cutoff Value; RFs = Remain Features; NoLs = Number of Labels;

Table S6. Results of sensitivity analysis (performance measured by Recall).

COP	COV	RFs	NoLs		LASSO																LASSO+SMOTE															
			0	1	AB	BC	DT	ET	GB	GNB	GP	KNN	LDA	LR	MLP	MNB	QDA	RF	SVM	XGB	AB	BC	DT	ET	GB	GNB	GP	KNN	LDA	LR	MLP	MNB	QDA	RF	SVM	XGB
54	1.28	1	193	141	0.67	0.67	0.67	0.67	0.67	0.67	0.67	0.67	0.67	0.67	0.67	0.00	0.67	0.67	0.67	0.67	0.67	0.67	0.67	0.67	0.67	0.67	0.67	0.67	0.67	0.67	0.67	0.00	0.67	0.67	0.67	0.67
54.5	1.28	1	193	141	0.67	0.67	0.67	0.67	0.67	0.67	0.67	0.67	0.67	0.67	0.67	0.00	0.67	0.67	0.67	0.67	0.67	0.67	0.67	0.67	0.67	0.67	0.67	0.67	0.67	0.67	0.67	0.00	0.67	0.67	0.67	0.67
55	1.28	1	193	141	0.67	0.67	0.67	0.67	0.67	0.67	0.67	0.67	0.67	0.67	0.67	0.00	0.67	0.67	0.67	0.67	0.67	0.67	0.67	0.67	0.67	0.67	0.67	0.67	0.67	0.67	0.67	0.00	0.67	0.67	0.67	0.67
55.5	1.28	1	193	141	0.67	0.67	0.67	0.67	0.67	0.67	0.67	0.67	0.67	0.67	0.67	0.00	0.67	0.67	0.67	0.67	0.67	0.67	0.67	0.67	0.67	0.67	0.67	0.67	0.67	0.67	0.67	0.00	0.67	0.67	0.67	0.67
56	1.28	1	193	141	0.67	0.67	0.67	0.67	0.67	0.67	0.67	0.67	0.67	0.67	0.67	0.00	0.67	0.67	0.67	0.67	0.67	0.67	0.67	0.67	0.67	0.67	0.67	0.67	0.67	0.67	0.67	0.00	0.67	0.67	0.67	0.67
56.5	3	57	199	135	0.50	0.81	0.82	0.72	0.67	0.65	0.78	0.90	0.66	0.60	0.61	0.73	0.76	0.68	0.76	0.81	0.63	0.61	0.40	0.56	0.48	0.70	0.79	0.74	0.60	0.63	0.39	0.77	0.82	0.70	0.72	0.69
57	3.24	57	202	132	0.54	0.75	0.44	0.71	0.60	0.60	0.85	0.54	0.56	0.75	0.70	0.67	0.80	0.73	0.68	0.70	0.55	0.77	0.86	0.71	0.71	0.67	0.77	0.67	0.62	0.60	0.59	0.63	0.71	0.60	0.77	0.83
57.5	3.24	63	202	132	0.54	0.75	0.44	0.71	0.60	0.60	0.85	0.54	0.56	0.75	0.70	0.67	0.80	0.73	0.68	0.70	0.55	0.77	0.86	0.71	0.71	0.67	0.77	0.67	0.62	0.60	0.59	0.63	0.71	0.60	0.77	0.83
58	3.24	63	202	132	0.54	0.75	0.44	0.71	0.60	0.60	0.85	0.54	0.56	0.75	0.70	0.67	0.80	0.73	0.68	0.70	0.55	0.77	0.86	0.71	0.71	0.67	0.77	0.67	0.62	0.60	0.59	0.63	0.71	0.60	0.77	0.83
58.5	5	63	209	125	0.70	0.58	0.58	0.54	0.66	0.70	0.78	0.53	0.60	0.70	0.54	0.54	0.53	0.77	0.79	0.76	0.62	0.50	0.67	0.61	0.65	0.66	0.67	0.68	0.77	0.59	0.68	0.66	0.86	0.66	0.71	0.76
59	5.88	63	212	122	0.56	0.75	0.51	0.49	0.66	0.73	0.43	0.84	0.57	0.62	0.54	0.65	0.59	0.68	0.35	0.68	0.74	0.90	0.79	0.63	0.60	0.72	0.34	0.78	0.67	0.66	0.68	0.63	0.70	0.72	0.71	0.75
59.5	5.88	57	212	122	0.56	0.75	0.51	0.49	0.66	0.73	0.43	0.84	0.57	0.62	0.54	0.65	0.59	0.68	0.35	0.68	0.74	0.90	0.79	0.63	0.60	0.72	0.34	0.78	0.67	0.66	0.68	0.63	0.70	0.72	0.71	0.75
60	7	57	213	121	0.67	0.81	0.78	0.66	0.84	0.73	0.79	0.86	0.55	0.74	0.46	0.62	0.62	0.45	0.68	0.71	0.75	0.58	0.79	0.64	0.57	0.73	0.75	0.69	0.67	0.69	0.71	0.78	0.76	0.71	0.74	0.56
60.5	8	62	214	120	0.64	0.58	0.83	0.72	0.74	0.63	0.43	0.52	0.62	0.76	0.37	0.69	0.53	0.61	0.59	0.65	0.74	0.48	0.48	0.89	0.79	0.74	0.64	0.59	0.73	0.75	0.72	0.68	0.74	0.72	0.73	0.70
61	8	67	214	120	0.64	0.58	0.83	0.72	0.74	0.63	0.43	0.52	0.62	0.76	0.37	0.69	0.53	0.61	0.59	0.65	0.74	0.48	0.48	0.89	0.79	0.74	0.64	0.59	0.73	0.75	0.72	0.68	0.74	0.72	0.73	0.70
61.5	9	61	215	119	0.48	0.72	0.42	0.60	0.72	0.67	0.79	0.88	0.50	0.67	0.76	0.61	0.72	0.71	0.85	0.61	0.63	0.55	0.46	0.52	0.71	0.66	0.61	0.73	0.73	0.71	0.55	0.69	0.73	0.57	0.66	0.51
62	10	61	216	118	0.63	0.58	0.69	0.69	0.64	0.73	0.78	0.88	0.47	0.57	0.79	0.54	0.55	0.52	0.81	0.83	0.62	0.64	0.53	0.64	0.75	0.72	0.81	0.67	0.63	0.64	0.69	0.88	0.56	0.63	0.59	0.59
62.5	11	55	217	117	0.62	0.62	0.78	0.94	0.91	0.66	0.44	0.47	0.66	0.59	0.53	0.64	0.67	0.71	0.62	0.79	0.61	0.67	0.57	0.85	0.86	0.74	0.73	0.71	0.56	0.59	0.62	0.84	0.38	0.82	0.56	0.50
63	12	55	218	116	0.63	0.61	0.59	0.69	0.53	0.78	0.73	0.72	0.63	0.70	0.53	0.59	0.75	0.62	0.79	0.67	0.63	0.59	0.45	0.45	0.86	0.73	0.72	0.37	0.65	0.62	0.69	0.70	0.80	0.74	0.62	0.65
63.5	13	63	221	113	0.35	0.64	0.56	0.77	0.55	0.81	0.77	0.82	0.52	0.66	0.72	0.68	0.53	0.66	0.69	0.50	0.64	0.50	0.56	0.86	0.67	0.73	0.65	0.73	0.57	0.62	0.74	0.43	0.72	0.88	0.64	0.73
64	14	60	222	112	0.40	0.47	0.39	0.71	0.70	0.77	0.73	0.49	0.71	0.65	0.79	0.65	0.73	0.62	0.79	0.82	0.65	0.73	0.42	0.60	0.59	0.70	0.61	0.80	0.63	0.69	0.68	0.88	0.70	0.77	0.54	0.54
64.5	14	56	222	112	0.40	0.47	0.39	0.71	0.70	0.77	0.73	0.49	0.71	0.65	0.79	0.65	0.73	0.62	0.79	0.82	0.65	0.73	0.42	0.60	0.59	0.70	0.61	0.80	0.63	0.69	0.68	0.88	0.70	0.77	0.54	0.54
65	16	53	226	108	0.44	0.57	0.66	0.71	0.65	0.74	0.80	0.76	0.47	0.53	0.69	0.81	0.58	0.45	0.84	0.77	0.63	0.88	0.56	0.91	0.61	0.73	0.70	0.57	0.71	0.72	0.72	0.71	0.63	0.81	0.66	0.85
65.5	18	56	229	105	0.69	0.51	0.38	0.62	0.61	0.77	0.61	0.60	0.66	0.74	0.70	0.39	0.39	0.49	0.73	0.70	0.72	0.78	0.95	0.73	0.67	0.73	0.70	0.63	0.52	0.62	0.80	0.38	0.53	0.69	0.73	0.90
66	19	57	230	104	0.56	0.55	0.39	0.78	0.50	0.76	0.64	0.60	0.61	0.74	0.57	0.70	0.45	0.61	0.75	0.58	0.69	0.58	0.78	0.78	0.82	0.70	0.62	0.71	0.57	0.75	0.65	0.38	0.60	0.69	0.71	0.53
66.5	20.78	56	232	102	0.68	0.78	0.42	0.73	0.74	0.75	0.81	0.75	0.59	0.56	0.69	0.82	0.54	0.80	0.69	0.71	0.62	0.62	0.31	0.74	0.58	0.62	0.59	0.63	0.69	0.66	0.62	0.83	0.63	0.79	0.74	0.87
67	22	56	233	101	0.57	0.71	0.53	0.71	0.79	0.84	0.77	0.89	0.56	0.64	0.63	0.76	0.59	0.75	0.80	0.76	0.65	0.65	0.50	0.84	0.57	0.77	0.45	0.80	0.58	0.71	0.61	0.81	0.74	0.83	0.82	0.89
67.5	23	34	235	99	0.58	0.76	0.34	0.70	0.62	0.76	0.75	0.77	0.58	0.65	0.64	0.63	0.70	0.68	0.75	0.87	0.64	0.57	0.64	0.83	0.61	0.61	0.72	0.91	0.62	0.59	0.65	0.66	0.64	0.68	0.62	0.82
68	24	37	236	98	0.55	0.49	0.44	0.71	0.63	0.77	0.87	0.74	0.59	0.64	0.59	0.79	0.74	0.70	0.80	0.82	0.54	0.63	0.50	0.80	0.65	0.87	0.58	0.41	0.50	0.57	0.92	0.82	0.64	0.89	0.70	0.69
68.5	26	68	239	95	0.43	0.93	0.35	0.88	0.48	0.76	0.88	0.72	0.66	0.54	0.60	0.62	0.79	0.81	0.84	0.62	0.43	0.61	0.87	0.65	0.84	0.87	0.72	0.76	0.47	0.74	0.77	0.78	0.74	0.74	0.89	0.86

69	27.08	39	241	93	0.51	0.70	0.46	0.68	0.86	0.69	0.89	0.57	0.58	0.60	0.75	0.70	0.75	0.62	0.83	0.85	0.49	0.46	0.91	0.78	0.70	0.80	0.42	0.70	0.53	0.66	0.61	0.77	0.61	0.73	0.62	0.76
69.5	29	36	243	91	0.58	0.93	0.33	0.66	0.45	0.79	0.82	0.69	0.54	0.53	0.76	0.67	0.90	0.52	0.84	0.68	0.67	0.86	0.57	0.78	0.78	0.85	0.85	0.65	0.46	0.65	0.54	0.79	0.74	0.77	0.46	0.62
70	31	36	246	88	0.61	0.63	0.45	0.72	0.90	0.78	0.84	0.67	0.59	0.77	0.48	0.68	0.81	0.88	0.90	0.64	0.59	0.69	0.82	0.67	0.70	0.86	0.58	0.58	0.74	0.67	0.76	0.63	0.66	0.85	0.70	0.74
70.5	32	37	249	85	0.47	0.71	0.51	0.85	0.56	0.68	0.84	0.80	0.51	0.68	0.69	0.65	0.80	0.86	0.85	0.82	0.64	0.76	0.75	0.59	0.87	0.60	0.58	0.71	0.72	0.68	0.56	0.80	0.81	0.73	0.60	0.68
71	34	60	253	81	0.53	0.53	0.58	0.81	0.64	0.79	0.83	0.63	0.26	0.54	0.72	0.83	0.40	0.77	0.86	0.51	0.53	0.51	0.73	0.58	0.77	0.68	0.60	0.59	0.70	0.57	0.51	0.62	0.69	0.77	0.68	0.64
71.5	36	59	254	80	0.56	0.73	0.33	0.66	0.83	0.78	0.83	0.90	0.46	0.63	0.41	0.80	0.70	0.76	0.89	0.79	0.65	0.56	0.98	0.74	0.71	0.65	0.76	0.34	0.66	0.65	0.71	0.51	0.71	0.85	0.64	0.59
72	37.04	39	256	78	0.81	0.76	0.33	0.62	0.77	0.65	0.91	0.85	0.38	0.68	0.60	0.64	0.77	0.65	0.86	0.86	0.76	0.82	0.87	0.63	0.88	0.83	0.81	0.67	0.73	0.68	0.92	0.64	0.64	0.73	0.47	0.86
72.5	40	38	259	75	0.71	0.68	0.56	0.69	0.95	0.64	0.89	0.83	0.77	0.93	0.75	0.60	0.71	0.76	0.92	0.67	0.65	0.75	0.53	0.83	0.72	0.53	0.80	0.84	0.80	0.85	0.91	0.59	0.67	0.84	0.65	0.67
73	42	44	259	75	0.57	0.75	0.51	0.57	0.48	0.67	0.92	0.59	0.53	0.49	0.97	0.65	0.87	0.60	0.63	0.88	0.95	0.89	0.49	0.89	0.65	0.39	0.69	0.59	0.59	0.63	0.59	0.72	0.81	0.91	0.57	0.75
73.5	45	38	260	74	0.73	0.41	0.39	0.88	0.74	0.86	0.77	0.89	0.53	0.77	0.81	0.32	0.62	0.80	0.78	0.77	0.61	0.77	0.78	0.68	0.68	0.64	0.65	0.50	0.62	0.53	0.51	0.58	0.74	0.77	0.93	0.88
74	50.36	37	261	73	0.58	0.66	0.33	0.90	0.93	0.71	0.64	0.84	0.78	0.70	0.71	0.42	0.77	0.32	0.93	0.88	0.74	0.64	0.95	0.74	0.78	0.81	0.79	0.47	0.84	0.71	0.66	0.63	0.82	0.90	0.71	0.89
74.5	53	46	262	72	0.33	0.94	0.24	0.76	0.58	0.74	0.83	0.96	0.40	0.74	0.38	0.49	0.82	0.79	0.94	0.68	0.71	0.65	0.90	0.83	0.76	0.85	0.72	0.53	0.74	0.74	0.60	0.72	0.78	0.81	0.42	0.79
75	55	44	263	71	0.66	0.63	0.55	0.68	0.93	0.82	0.46	0.79	0.49	0.69	0.85	0.76	0.65	0.87	0.76	0.80	0.61	0.87	0.70	0.82	0.61	0.59	0.59	0.55	0.63	0.61	0.62	0.59	0.86	0.73	0.61	0.70
75.5	59	35	265	69	0.84	0.87	0.30	0.90	0.90	0.86	0.75	0.62	0.87	0.71	0.81	0.91	0.32	0.88	0.72	0.35	0.87	0.71	0.70	0.71	0.58	0.71	0.72	0.81	0.42	0.55	0.72	0.49	0.80	0.61	0.71	0.49
76	63	38	266	68	0.87	0.38	0.34	0.91	0.37	0.94	0.63	0.62	0.88	0.53	0.60	0.85	0.91	0.85	0.88	0.57	0.57	0.66	0.88	0.63	0.63	0.76	0.38	0.62	0.56	0.78	0.84	0.68	0.57	0.82	0.78	0.41
76.5	66	42	267	67	0.69	0.60	0.48	0.63	0.60	0.84	0.66	0.67	0.78	0.82	0.48	0.90	0.88	0.60	0.67	0.63	0.69	0.54	0.99	0.84	0.76	0.64	0.58	0.49	0.64	0.70	0.69	0.45	0.64	0.75	0.52	0.60
77	70	39	268	66	0.85	0.64	0.41	0.88	0.89	0.74	0.64	0.73	0.83	0.74	0.59	0.95	0.85	0.94	0.53	0.64	0.64	0.42	0.79	0.52	0.65	0.59	0.92	0.55	0.58	0.71	0.77	0.71	0.59	0.73	0.80	0.56
77.5	74	42	270	64	0.75	0.45	0.47	0.48	0.88	0.91	0.70	0.66	0.81	0.59	0.47	0.95	0.81	0.78	0.97	0.66	0.63	0.47	0.42	0.63	0.67	0.72	0.34	0.61	0.72	0.64	0.80	0.75	0.69	0.69	0.63	0.59
78	80	38	271	63	0.37	0.38	0.59	0.29	0.57	0.95	0.44	0.63	0.89	0.52	0.25	0.30	0.29	0.41	0.65	0.54	0.56	0.83	0.87	0.86	0.70	0.60	0.68	0.79	0.75	0.81	0.78	0.78	0.68	0.81	0.35	0.60
78.5	86	38	271	63	0.44	0.43	0.49	0.63	0.29	0.84	0.59	0.60	0.40	0.79	0.29	0.81	0.35	0.75	0.89	0.59	0.54	0.65	0.95	0.71	0.83	0.59	0.63	0.52	0.84	0.73	0.81	0.75	0.76	0.79	0.79	0.71
79	90.28	43	273	61	0.39	0.59	0.49	0.36	0.87	0.80	0.69	0.77	0.39	0.57	0.69	0.77	0.82	0.90	0.95	0.95	0.54	0.75	0.57	0.36	0.61	0.54	0.90	0.74	0.61	0.61	0.84	0.43	0.67	0.74	0.77	0.74
79.5	94	43	275	59	0.37	0.85	0.37	0.88	0.20	0.24	0.71	0.32	0.42	0.61	0.97	0.25	0.86	0.76	0.81	0.92	0.59	0.71	0.63	0.78	0.88	0.68	0.42	0.56	0.61	0.59	0.53	0.68	0.69	0.68	0.76	0.92
80	101.6	34	275	59	0.25	0.78	0.42	0.54	0.41	0.39	0.69	0.53	0.29	0.76	0.49	0.80	0.32	0.71	0.81	0.64	0.71	0.54	0.83	0.86	0.68	0.64	0.73	0.76	0.73	0.76	0.88	0.66	0.75	0.78	0.78	0.59
80.5	107	31	277	57	0.63	0.67	0.63	0.63	0.56	0.28	0.65	0.53	0.56	0.75	0.56	0.54	0.30	0.54	0.65	0.75	0.72	0.86	0.35	0.81	0.86	0.44	0.77	0.51	0.75	0.81	0.68	0.74	0.86	0.74	0.44	0.46
81	111.92	42	278	56	0.52	0.32	0.57	0.71	0.61	0.34	0.63	0.61	0.96	0.82	0.63	0.79	0.36	0.73	0.61	0.64	0.68	0.80	0.96	0.79	0.80	0.41	0.68	0.57	0.63	0.77	0.77	0.52	0.77	0.77	0.45	0.64
81.5	120.58	42	280	54	0.44	0.35	0.31	0.61	0.39	0.28	0.50	0.83	0.30	0.72	0.50	0.78	0.33	0.56	0.80	0.65	0.78	0.70	0.85	0.56	0.80	0.43	0.72	0.72	0.74	0.76	0.44	0.74	0.65	0.78	0.85	0.63
82	131.24	41	281	53	0.55	0.26	0.53	0.55	0.34	0.77	0.66	0.47	0.77	0.83	0.70	0.66	0.28	0.55	0.21	0.53	0.79	0.60	0.94	0.75	0.77	0.49	0.64	0.60	0.62	0.75	0.75	0.62	0.81	0.85	0.58	0.55
82.5	141	38	281	53	0.60	0.60	0.30	0.60	0.38	0.25	0.53	0.26	0.92	0.70	0.30	0.74	0.25	0.47	0.68	0.43	0.77	0.47	0.89	0.77	0.75	0.58	0.92	0.75	0.75	0.70	0.74	0.66	0.79	0.79	0.53	0.34
83	150	40	282	52	0.79	0.19	0.33	0.60	0.27	0.83	0.38	0.33	0.77	0.69	0.48	0.73	0.27	0.40	0.38	0.29	0.75	0.46	0.85	0.81	0.75	0.65	0.90	0.60	0.69	0.73	0.60	0.71	0.81	0.71	0.81	0.67
83.5	157.22	44	282	52	0.79	0.21	0.48	0.31	0.12	0.65	0.46	0.65	0.67	0.75	0.48	0.69	0.31	0.25	0.60	0.19	0.73	0.58	0.92	0.73	0.50	0.60	0.81	0.92	0.71	0.75	0.87	0.73	0.54	0.77	0.77	0.83
84	167.88	22	284	50	0.70	0.34	0.50	0.54	0.92	0.70	0.94	0.58	0.94	0.86	0.82	0.74	0.82	0.80	1.00	0.82	0.74	0.80	0.82	0.50	0.70	0.68	0.72	0.92	0.74	0.74	0.84	0.78	0.78	0.86	0.30	0.84
84.5	175.54	21	284	50	0.86	0.60	0.56	0.66	0.64	0.38	0.82	0.54	0.82	0.88	0.90	0.56	0.88	0.72	0.80	0.58	0.78	0.90	0.84	0.86	0.90	0.72	0.74	0.94	0.74	0.80	0.92	0.72	0.78	0.86	0.64	0.62
85	185.2	13	286	48	0.90	0.94	0.77	0.92	0.65	0.73	0.94	0.83	0.90	0.85	0.73	0.63	0.83	0.94	0.77	0.23	0.83	0.81	0.98	0.83	0.92	0.60	0.88	0.13	0.83	0.83	0.83	0.71	0.85	0.88	0.96	0.69

Abbreviation: COP = Cutoff Percentile; COV = Cutoff Value; RFs = Remain Features; NoLs = Number of Labels;

Table S7. Results of sensitivity analysis (performance measured by Precision).

COP	COV	RFs	NoLs		LASSO																LASSO+SMOTE															
			0	1	AB	BC	DT	ET	GB	GNB	GP	KNN	LDA	LR	MLP	MNB	QDA	RF	SVM	XGB	AB	BC	DT	ET	GB	GNB	GP	KNN	LDA	LR	MLP	MNB	QDA	RF	SVM	XGB
54	1.28	1	193	141	0.45	0.45	0.45	0.45	0.45	0.45	0.45	0.45	0.45	0.45	0.45	0.00	0.45	0.45	0.45	0.45	0.45	0.45	0.45	0.45	0.45	0.45	0.45	0.45	0.45	0.45	0.45	0.00	0.45	0.45	0.45	0.45
54.5	1.28	1	193	141	0.45	0.45	0.45	0.45	0.45	0.45	0.45	0.45	0.45	0.45	0.45	0.00	0.45	0.45	0.45	0.45	0.45	0.45	0.45	0.45	0.45	0.45	0.45	0.45	0.45	0.45	0.45	0.00	0.45	0.45	0.45	0.45
55	1.28	1	193	141	0.45	0.45	0.45	0.45	0.45	0.45	0.45	0.45	0.45	0.45	0.45	0.00	0.45	0.45	0.45	0.45	0.45	0.45	0.45	0.45	0.45	0.45	0.45	0.45	0.45	0.45	0.45	0.00	0.45	0.45	0.45	0.45
55.5	1.28	1	193	141	0.45	0.45	0.45	0.45	0.45	0.45	0.45	0.45	0.45	0.45	0.45	0.00	0.45	0.45	0.45	0.45	0.45	0.45	0.45	0.45	0.45	0.45	0.45	0.45	0.45	0.45	0.45	0.00	0.45	0.45	0.45	0.45
56	1.28	1	193	141	0.45	0.45	0.45	0.45	0.45	0.45	0.45	0.45	0.45	0.45	0.45	0.00	0.45	0.45	0.45	0.45	0.45	0.45	0.45	0.45	0.45	0.45	0.45	0.45	0.45	0.45	0.45	0.00	0.45	0.45	0.45	0.45
56.5	3	57	199	135	0.57	0.47	0.43	0.50	0.56	0.49	0.48	0.44	0.52	0.55	0.54	0.51	0.53	0.49	0.47	0.50	0.54	0.51	0.47	0.59	0.61	0.51	0.48	0.46	0.56	0.55	0.67	0.49	0.52	0.54	0.55	0.55
57	3.24	57	202	132	0.58	0.48	0.43	0.51	0.54	0.53	0.49	0.45	0.54	0.53	0.52	0.50	0.52	0.49	0.44	0.54	0.59	0.53	0.43	0.53	0.53	0.55	0.48	0.52	0.56	0.56	0.59	0.52	0.55	0.56	0.52	0.51
57.5	3.24	63	202	132	0.58	0.48	0.43	0.51	0.54	0.53	0.49	0.45	0.54	0.53	0.52	0.50	0.52	0.49	0.44	0.54	0.59	0.53	0.43	0.53	0.53	0.55	0.48	0.52	0.56	0.56	0.59	0.52	0.55	0.56	0.52	0.51
58	3.24	63	202	132	0.58	0.48	0.43	0.51	0.54	0.53	0.49	0.45	0.54	0.53	0.52	0.50	0.52	0.49	0.44	0.54	0.59	0.53	0.43	0.53	0.53	0.55	0.48	0.52	0.56	0.56	0.59	0.52	0.55	0.56	0.52	0.51
58.5	5	63	209	125	0.50	0.45	0.42	0.54	0.50	0.48	0.46	0.43	0.55	0.52	0.61	0.52	0.58	0.50	0.42	0.50	0.55	0.54	0.42	0.53	0.51	0.53	0.44	0.50	0.50	0.54	0.52	0.49	0.47	0.55	0.52	0.51
59	5.88	63	212	122	0.55	0.48	0.45	0.55	0.51	0.46	0.51	0.40	0.55	0.55	0.57	0.47	0.52	0.47	0.46	0.52	0.51	0.44	0.40	0.56	0.56	0.50	0.57	0.45	0.53	0.53	0.52	0.48	0.49	0.52	0.52	0.48
59.5	5.88	57	212	122	0.55	0.48	0.45	0.55	0.51	0.46	0.51	0.40	0.55	0.55	0.57	0.47	0.52	0.47	0.46	0.52	0.51	0.44	0.40	0.56	0.56	0.50	0.57	0.45	0.53	0.53	0.52	0.48	0.49	0.52	0.52	0.48
60	7	57	213	121	0.47	0.47	0.39	0.48	0.46	0.44	0.43	0.37	0.53	0.51	0.62	0.46	0.47	0.56	0.42	0.50	0.49	0.50	0.40	0.49	0.56	0.48	0.43	0.46	0.51	0.52	0.49	0.44	0.48	0.48	0.48	0.53
60.5	8	62	214	120	0.48	0.48	0.40	0.48	0.49	0.46	0.48	0.44	0.51	0.48	0.58	0.47	0.56	0.53	0.47	0.52	0.48	0.49	0.46	0.45	0.49	0.48	0.44	0.44	0.50	0.50	0.48	0.45	0.49	0.50	0.49	0.48
61	8	67	214	120	0.48	0.48	0.40	0.48	0.49	0.46	0.48	0.44	0.51	0.48	0.58	0.47	0.56	0.53	0.47	0.52	0.48	0.49	0.46	0.45	0.49	0.48	0.44	0.44	0.50	0.50	0.48	0.45	0.49	0.50	0.49	0.48
61.5	9	61	215	119	0.46	0.42	0.45	0.49	0.46	0.41	0.42	0.39	0.49	0.50	0.45	0.42	0.43	0.45	0.40	0.49	0.51	0.51	0.46	0.52	0.47	0.43	0.44	0.46	0.49	0.48	0.54	0.42	0.48	0.50	0.50	0.56
62	10	61	216	118	0.44	0.43	0.39	0.42	0.46	0.40	0.43	0.38	0.53	0.52	0.41	0.44	0.46	0.46	0.38	0.43	0.50	0.48	0.43	0.47	0.47	0.43	0.40	0.43	0.50	0.50	0.47	0.40	0.50	0.48	0.50	0.51
62.5	11	55	217	117	0.50	0.46	0.41	0.41	0.42	0.42	0.46	0.43	0.48	0.53	0.53	0.42	0.44	0.44	0.41	0.45	0.52	0.47	0.45	0.44	0.44	0.44	0.42	0.44	0.51	0.53	0.53	0.40	0.61	0.45	0.53	0.57
63	12	55	218	116	0.43	0.46	0.38	0.43	0.48	0.40	0.40	0.38	0.47	0.49	0.42	0.42	0.41	0.44	0.39	0.46	0.50	0.46	0.44	0.49	0.43	0.42	0.41	0.55	0.50	0.50	0.45	0.41	0.43	0.45	0.48	0.47
63.5	13	63	221	113	0.51	0.47	0.38	0.42	0.52	0.40	0.40	0.42	0.46	0.47	0.42	0.39	0.44	0.46	0.41	0.50	0.48	0.49	0.43	0.42	0.45	0.41	0.44	0.42	0.50	0.51	0.45	0.45	0.44	0.41	0.44	0.45
64	14	60	222	112	0.49	0.46	0.37	0.42	0.45	0.38	0.42	0.40	0.41	0.47	0.44	0.39	0.40	0.46	0.40	0.42	0.46	0.41	0.48	0.50	0.48	0.41	0.42	0.37	0.49	0.46	0.48	0.39	0.43	0.43	0.48	0.51
64.5	14	56	222	112	0.49	0.46	0.37	0.42	0.45	0.38	0.42	0.40	0.41	0.47	0.44	0.39	0.40	0.46	0.40	0.42	0.46	0.41	0.48	0.50	0.48	0.41	0.42	0.37	0.49	0.46	0.48	0.39	0.43	0.43	0.48	0.51
65	16	53	226	108	0.46	0.41	0.40	0.44	0.41	0.37	0.38	0.38	0.45	0.52	0.41	0.38	0.41	0.51	0.39	0.41	0.47	0.40	0.42	0.41	0.52	0.41	0.39	0.44	0.46	0.46	0.44	0.39	0.44	0.42	0.43	0.41
65.5	18	56	229	105	0.40	0.47	0.33	0.42	0.42	0.37	0.39	0.43	0.41	0.43	0.38	0.43	0.46	0.43	0.39	0.45	0.42	0.39	0.32	0.41	0.46	0.39	0.41	0.40	0.47	0.46	0.41	0.50	0.47	0.45	0.43	0.39
66	19	57	230	104	0.43	0.43	0.38	0.37	0.46	0.37	0.36	0.40	0.40	0.42	0.46	0.37	0.41	0.38	0.36	0.42	0.45	0.42	0.34	0.40	0.40	0.38	0.40	0.38	0.46	0.43	0.46	0.45	0.44	0.44	0.43	0.47
66.5	20.78	56	232	102	0.39	0.38	0.41	0.38	0.40	0.36	0.36	0.37	0.41	0.46	0.43	0.35	0.42	0.38	0.39	0.43	0.44	0.41	0.36	0.41	0.45	0.40	0.40	0.45	0.43	0.44	0.43	0.35	0.44	0.40	0.41	0.43
67	22	56	233	101	0.41	0.39	0.35	0.38	0.39	0.35	0.37	0.36	0.41	0.44	0.42	0.35	0.40	0.39	0.39	0.43	0.43	0.41	0.37	0.38	0.48	0.37	0.45	0.41	0.45	0.44	0.43	0.35	0.41	0.41	0.39	0.39
67.5	23	34	235	99	0.42	0.36	0.34	0.37	0.45	0.35	0.35	0.32	0.39	0.41	0.42	0.35	0.38	0.37	0.37	0.38	0.45	0.40	0.35	0.38	0.45	0.40	0.36	0.35	0.45	0.46	0.45	0.36	0.44	0.42	0.41	0.40
68	24	37	236	98	0.42	0.42	0.34	0.41	0.41	0.36	0.33	0.37	0.39	0.43	0.44	0.35	0.37	0.39	0.37	0.39	0.48	0.45	0.40	0.38	0.44	0.38	0.39	0.45	0.48	0.44	0.38	0.36	0.46	0.38	0.40	0.46
68.5	26	68	239	95	0.41	0.35	0.33	0.36	0.42	0.33	0.33	0.36	0.35	0.44	0.43	0.32	0.35	0.36	0.34	0.41	0.53	0.45	0.32	0.43	0.40	0.35	0.37	0.36	0.46	0.37	0.38	0.35	0.39	0.40	0.35	0.38
69	27.08	39	241	93	0.42	0.36	0.35	0.36	0.36	0.33	0.33	0.37	0.39	0.40	0.39	0.33	0.34	0.36	0.32	0.39	0.45	0.45	0.30	0.37	0.40	0.36	0.42	0.35	0.44	0.41	0.40	0.36	0.44	0.41	0.41	0.39

69.5	29	36	243	91	0.38	0.33	0.30	0.37	0.43	0.32	0.32	0.35	0.37	0.44	0.40	0.34	0.33	0.40	0.32	0.43	0.39	0.38	0.38	0.37	0.38	0.34	0.32	0.37	0.46	0.38	0.42	0.35	0.44	0.39	0.41	0.42
70	31	36	246	88	0.35	0.33	0.27	0.31	0.32	0.30	0.30	0.32	0.36	0.36	0.41	0.31	0.31	0.31	0.29	0.37	0.42	0.38	0.29	0.40	0.39	0.34	0.36	0.36	0.38	0.37	0.35	0.36	0.40	0.34	0.36	0.37
70.5	32	37	249	85	0.35	0.32	0.29	0.32	0.37	0.30	0.28	0.29	0.32	0.38	0.38	0.31	0.30	0.31	0.29	0.35	0.38	0.38	0.28	0.40	0.36	0.39	0.36	0.31	0.34	0.36	0.38	0.33	0.37	0.35	0.41	0.41
71	34	60	253	81	0.35	0.38	0.31	0.31	0.35	0.28	0.29	0.32	0.48	0.40	0.32	0.28	0.37	0.32	0.30	0.37	0.39	0.43	0.31	0.42	0.34	0.34	0.33	0.37	0.35	0.41	0.42	0.34	0.39	0.34	0.35	0.34
71.5	36	59	254	80	0.33	0.32	0.27	0.32	0.28	0.27	0.28	0.30	0.39	0.39	0.34	0.27	0.29	0.30	0.29	0.32	0.40	0.37	0.27	0.34	0.34	0.35	0.35	0.37	0.36	0.36	0.35	0.37	0.39	0.32	0.35	0.35
72	37.04	39	256	78	0.28	0.31	0.29	0.36	0.30	0.29	0.28	0.30	0.36	0.34	0.35	0.28	0.28	0.32	0.29	0.32	0.32	0.36	0.27	0.36	0.31	0.30	0.30	0.35	0.34	0.35	0.30	0.32	0.40	0.36	0.41	0.33
72.5	40	38	259	75	0.27	0.30	0.27	0.31	0.27	0.27	0.25	0.26	0.27	0.30	0.28	0.29	0.28	0.30	0.27	0.33	0.35	0.35	0.34	0.35	0.31	0.38	0.28	0.30	0.30	0.30	0.28	0.32	0.37	0.30	0.34	0.35
73	42	44	259	75	0.30	0.29	0.27	0.33	0.35	0.28	0.26	0.31	0.31	0.43	0.26	0.29	0.27	0.34	0.28	0.31	0.29	0.30	0.26	0.30	0.33	0.41	0.30	0.32	0.36	0.35	0.33	0.30	0.32	0.32	0.39	0.31
73.5	45	38	260	74	0.27	0.29	0.31	0.26	0.29	0.25	0.26	0.26	0.29	0.31	0.28	0.33	0.28	0.26	0.26	0.27	0.34	0.32	0.26	0.32	0.32	0.29	0.28	0.28	0.35	0.39	0.38	0.32	0.32	0.32	0.29	0.27
74	50.36	37	261	73	0.29	0.26	0.27	0.28	0.27	0.26	0.26	0.26	0.26	0.32	0.28	0.30	0.27	0.35	0.25	0.26	0.32	0.31	0.24	0.30	0.28	0.28	0.27	0.31	0.31	0.35	0.32	0.29	0.34	0.30	0.32	0.30
74.5	53	46	262	72	0.34	0.25	0.22	0.27	0.28	0.26	0.26	0.24	0.33	0.30	0.30	0.27	0.25	0.26	0.25	0.30	0.32	0.31	0.25	0.29	0.32	0.27	0.28	0.31	0.32	0.32	0.30	0.28	0.31	0.31	0.42	0.29
75	55	44	263	71	0.27	0.28	0.25	0.30	0.26	0.25	0.27	0.23	0.28	0.32	0.25	0.25	0.27	0.27	0.26	0.30	0.35	0.28	0.33	0.29	0.38	0.30	0.30	0.29	0.33	0.34	0.34	0.29	0.31	0.33	0.34	0.32
75.5	59	35	265	69	0.24	0.26	0.21	0.24	0.25	0.24	0.24	0.27	0.23	0.30	0.25	0.23	0.33	0.25	0.25	0.32	0.28	0.30	0.23	0.34	0.36	0.27	0.27	0.25	0.41	0.34	0.30	0.31	0.29	0.32	0.27	0.33
76	63	38	266	68	0.25	0.31	0.24	0.24	0.35	0.23	0.26	0.26	0.24	0.38	0.28	0.24	0.25	0.24	0.24	0.27	0.36	0.33	0.23	0.34	0.33	0.27	0.37	0.26	0.37	0.29	0.29	0.29	0.34	0.28	0.26	0.35
76.5	66	42	267	67	0.24	0.29	0.24	0.27	0.26	0.24	0.27	0.23	0.23	0.28	0.26	0.24	0.24	0.27	0.27	0.26	0.30	0.31	0.22	0.26	0.32	0.29	0.29	0.34	0.32	0.31	0.32	0.35	0.35	0.30	0.32	0.33
77	70	39	268	66	0.24	0.25	0.29	0.24	0.23	0.24	0.26	0.22	0.23	0.30	0.25	0.23	0.24	0.23	0.26	0.26	0.31	0.33	0.24	0.33	0.29	0.30	0.23	0.32	0.33	0.28	0.29	0.27	0.35	0.28	0.25	0.31
77.5	74	42	270	64	0.24	0.29	0.22	0.27	0.23	0.22	0.24	0.26	0.23	0.32	0.32	0.22	0.24	0.24	0.22	0.26	0.33	0.32	0.33	0.31	0.30	0.29	0.32	0.32	0.27	0.28	0.28	0.25	0.30	0.29	0.27	0.26
78	80	38	271	63	0.31	0.33	0.26	0.35	0.26	0.22	0.27	0.24	0.22	0.32	0.39	0.33	0.35	0.27	0.22	0.27	0.34	0.30	0.22	0.26	0.31	0.29	0.24	0.24	0.28	0.27	0.28	0.26	0.32	0.26	0.29	0.27
78.5	86	38	271	63	0.27	0.28	0.23	0.26	0.31	0.22	0.26	0.23	0.27	0.27	0.33	0.22	0.30	0.25	0.21	0.23	0.32	0.26	0.21	0.28	0.26	0.28	0.25	0.32	0.27	0.29	0.26	0.26	0.30	0.28	0.25	0.26
79	90.28	43	273	61	0.27	0.24	0.23	0.28	0.22	0.22	0.22	0.22	0.26	0.32	0.25	0.22	0.22	0.21	0.21	0.21	0.33	0.25	0.23	0.34	0.28	0.30	0.22	0.25	0.32	0.33	0.26	0.38	0.34	0.26	0.24	0.25
79.5	94	43	275	59	0.26	0.21	0.20	0.21	0.32	0.33	0.21	0.25	0.23	0.30	0.20	0.33	0.20	0.22	0.22	0.21	0.32	0.25	0.22	0.25	0.24	0.28	0.31	0.26	0.29	0.32	0.31	0.25	0.27	0.27	0.23	0.23
80	101.6	34	275	59	0.31	0.22	0.26	0.26	0.29	0.28	0.22	0.24	0.31	0.27	0.23	0.20	0.32	0.23	0.21	0.24	0.29	0.30	0.20	0.23	0.30	0.26	0.24	0.25	0.28	0.29	0.23	0.25	0.29	0.27	0.20	0.26
80.5	107	31	277	57	0.21	0.23	0.23	0.24	0.24	0.36	0.25	0.21	0.22	0.25	0.25	0.21	0.28	0.23	0.22	0.23	0.27	0.20	0.24	0.21	0.25	0.32	0.23	0.32	0.27	0.25	0.27	0.23	0.23	0.23	0.22	0.30
81	111.92	42	278	56	0.22	0.33	0.25	0.22	0.26	0.28	0.24	0.22	0.19	0.26	0.24	0.20	0.30	0.22	0.22	0.25	0.28	0.26	0.19	0.26	0.27	0.35	0.23	0.26	0.30	0.29	0.23	0.28	0.28	0.25	0.27	0.27
81.5	120.58	42	280	54	0.22	0.28	0.22	0.22	0.27	0.31	0.24	0.19	0.24	0.27	0.22	0.20	0.26	0.22	0.20	0.23	0.26	0.25	0.20	0.28	0.23	0.34	0.23	0.23	0.28	0.26	0.32	0.22	0.28	0.22	0.22	0.28
82	131.24	41	281	53	0.20	0.37	0.20	0.22	0.25	0.19	0.23	0.22	0.18	0.24	0.21	0.20	0.32	0.22	0.38	0.22	0.26	0.27	0.19	0.22	0.25	0.33	0.23	0.25	0.26	0.27	0.26	0.27	0.25	0.21	0.24	0.26
82.5	141	38	281	53	0.20	0.22	0.20	0.22	0.23	0.35	0.21	0.25	0.18	0.28	0.23	0.20	0.34	0.22	0.19	0.23	0.27	0.28	0.19	0.20	0.23	0.30	0.21	0.21	0.31	0.26	0.27	0.27	0.26	0.24	0.21	0.27
83	150	40	282	52	0.19	0.40	0.25	0.22	0.34	0.19	0.25	0.28	0.19	0.27	0.21	0.21	0.41	0.30	0.23	0.29	0.29	0.27	0.18	0.21	0.25	0.27	0.21	0.22	0.28	0.26	0.30	0.27	0.28	0.28	0.23	0.21
83.5	157.22	44	282	52	0.20	0.39	0.19	0.29	0.43	0.21	0.22	0.19	0.19	0.25	0.20	0.20	0.27	0.33	0.19	0.32	0.27	0.27	0.17	0.24	0.31	0.33	0.21	0.18	0.27	0.26	0.23	0.27	0.32	0.23	0.20	0.20
84	167.88	22	284	50	0.19	0.27	0.23	0.24	0.17	0.20	0.17	0.21	0.18	0.23	0.17	0.20	0.18	0.18	0.17	0.18	0.28	0.21	0.17	0.35	0.23	0.26	0.21	0.17	0.29	0.28	0.22	0.27	0.27	0.23	0.31	0.18
84.5	175.54	21	284	50	0.18	0.24	0.23	0.23	0.24	0.25	0.20	0.24	0.19	0.20	0.19	0.20	0.18	0.24	0.21	0.25	0.21	0.20	0.18	0.20	0.20	0.21	0.22	0.18	0.21	0.21	0.19	0.22	0.21	0.20	0.18	0.22
85	185.2	13	286	48	0.17	0.19	0.17	0.17	0.19	0.19	0.17	0.20	0.17	0.19	0.19	0.20	0.18	0.19	0.18	0.32	0.19	0.21	0.16	0.19	0.18	0.21	0.19	0.46	0.19	0.19	0.19	0.20	0.18	0.20	0.16	0.19

Abbreviation: COP = Cutoff Percentile; COV = Cutoff Value; RFs = Remain Features; NoLs = Number of Labels;

Table S8. Results of sensitivity analysis (performance measured by F1-Score).

COP	COV	RFs	NoLs		LASSO																LASSO+SMOTE															
			0	1	AB	BC	DT	ET	GB	GNB	GP	KNN	LDA	LR	MLP	MNB	QDA	RF	SVM	XGB	AB	BC	DT	ET	GB	GNB	GP	KNN	LDA	LR	MLP	MNB	QDA	RF	SVM	XGB
54	1.28	1	193	141	0.54	0.54	0.54	0.54	0.54	0.54	0.54	0.45	0.54	0.54	0.54	0.00	0.54	0.54	0.54	0.54	0.54	0.54	0.54	0.54	0.54	0.54	0.54	0.54	0.54	0.54	0.54	0.00	0.54	0.54	0.54	0.54
54.5	1.28	1	193	141	0.54	0.54	0.54	0.54	0.54	0.54	0.54	0.45	0.54	0.54	0.54	0.00	0.54	0.54	0.54	0.54	0.54	0.54	0.54	0.54	0.54	0.54	0.54	0.54	0.54	0.54	0.54	0.00	0.54	0.54	0.54	0.54
55	1.28	1	193	141	0.54	0.54	0.54	0.54	0.54	0.54	0.54	0.45	0.54	0.54	0.54	0.00	0.54	0.54	0.54	0.54	0.54	0.54	0.54	0.54	0.54	0.54	0.54	0.54	0.54	0.54	0.54	0.00	0.54	0.54	0.54	0.54
55.5	1.28	1	193	141	0.54	0.54	0.54	0.54	0.54	0.54	0.54	0.45	0.54	0.54	0.54	0.00	0.54	0.54	0.54	0.54	0.54	0.54	0.54	0.54	0.54	0.54	0.54	0.54	0.54	0.54	0.54	0.00	0.54	0.54	0.54	0.54
56	1.28	1	193	141	0.54	0.54	0.54	0.54	0.54	0.54	0.54	0.45	0.54	0.54	0.54	0.00	0.54	0.54	0.54	0.54	0.54	0.54	0.54	0.54	0.54	0.54	0.54	0.54	0.54	0.54	0.54	0.00	0.54	0.54	0.54	0.54
56.5	3	57	199	135	0.53	0.60	0.57	0.59	0.61	0.56	0.59	0.44	0.58	0.58	0.57	0.60	0.62	0.57	0.58	0.62	0.58	0.55	0.43	0.58	0.54	0.59	0.59	0.57	0.58	0.59	0.50	0.60	0.64	0.61	0.62	0.61
57	3.24	57	202	132	0.56	0.59	0.44	0.60	0.57	0.56	0.62	0.45	0.55	0.62	0.60	0.57	0.63	0.59	0.54	0.61	0.57	0.62	0.57	0.61	0.60	0.61	0.59	0.59	0.59	0.58	0.59	0.57	0.62	0.58	0.62	0.64
57.5	3.24	63	202	132	0.56	0.59	0.44	0.60	0.57	0.56	0.62	0.45	0.55	0.62	0.60	0.57	0.63	0.59	0.54	0.61	0.57	0.62	0.57	0.61	0.60	0.61	0.59	0.59	0.59	0.58	0.59	0.57	0.62	0.58	0.62	0.64
58	3.24	63	202	132	0.56	0.59	0.44	0.60	0.57	0.56	0.62	0.45	0.55	0.62	0.60	0.57	0.63	0.59	0.54	0.61	0.57	0.62	0.57	0.61	0.60	0.61	0.59	0.59	0.59	0.58	0.59	0.57	0.62	0.58	0.62	0.64
58.5	5	63	209	125	0.58	0.51	0.49	0.54	0.57	0.57	0.58	0.43	0.57	0.60	0.57	0.53	0.55	0.60	0.55	0.60	0.58	0.52	0.52	0.57	0.57	0.59	0.54	0.58	0.61	0.56	0.59	0.56	0.61	0.60	0.60	0.61
59	5.88	63	212	122	0.56	0.58	0.48	0.52	0.57	0.56	0.46	0.40	0.56	0.58	0.56	0.54	0.55	0.56	0.40	0.59	0.60	0.59	0.53	0.59	0.58	0.59	0.43	0.57	0.59	0.59	0.59	0.55	0.57	0.61	0.60	0.59
59.5	5.88	57	212	122	0.56	0.58	0.48	0.52	0.57	0.56	0.46	0.40	0.56	0.58	0.56	0.54	0.55	0.56	0.40	0.59	0.60	0.59	0.53	0.59	0.58	0.59	0.43	0.57	0.59	0.59	0.59	0.55	0.57	0.61	0.60	0.59
60	7	57	213	121	0.55	0.60	0.52	0.56	0.60	0.55	0.56	0.37	0.54	0.60	0.53	0.53	0.54	0.50	0.52	0.59	0.59	0.54	0.53	0.56	0.56	0.58	0.54	0.55	0.58	0.59	0.58	0.56	0.59	0.58	0.58	0.54
60.5	8	62	214	120	0.55	0.53	0.54	0.57	0.59	0.53	0.45	0.44	0.56	0.59	0.45	0.56	0.55	0.56	0.52	0.58	0.58	0.48	0.47	0.60	0.61	0.58	0.52	0.51	0.59	0.60	0.58	0.54	0.59	0.59	0.58	0.57
61	8	67	214	120	0.55	0.53	0.54	0.57	0.59	0.53	0.45	0.44	0.56	0.59	0.45	0.56	0.55	0.56	0.52	0.58	0.58	0.48	0.47	0.60	0.61	0.58	0.52	0.51	0.59	0.60	0.58	0.54	0.59	0.59	0.58	0.57
61.5	9	61	215	119	0.47	0.53	0.43	0.54	0.56	0.51	0.55	0.39	0.50	0.58	0.56	0.50	0.54	0.56	0.54	0.55	0.57	0.53	0.46	0.52	0.56	0.52	0.51	0.56	0.59	0.57	0.55	0.52	0.58	0.53	0.57	0.54
62	10	61	216	118	0.52	0.50	0.50	0.52	0.54	0.52	0.55	0.38	0.50	0.54	0.54	0.49	0.50	0.48	0.52	0.57	0.55	0.55	0.47	0.54	0.58	0.54	0.53	0.52	0.56	0.56	0.56	0.55	0.53	0.54	0.54	0.55
62.5	11	55	217	117	0.55	0.53	0.54	0.57	0.57	0.51	0.45	0.43	0.56	0.56	0.53	0.51	0.53	0.54	0.49	0.58	0.56	0.55	0.50	0.58	0.58	0.55	0.53	0.55	0.53	0.56	0.57	0.55	0.47	0.58	0.54	0.53
63	12	55	218	116	0.51	0.53	0.46	0.53	0.50	0.53	0.52	0.38	0.54	0.57	0.47	0.49	0.53	0.51	0.52	0.54	0.56	0.52	0.45	0.47	0.57	0.53	0.52	0.44	0.57	0.55	0.54	0.51	0.56	0.56	0.54	0.54
63.5	13	63	221	113	0.41	0.54	0.45	0.54	0.53	0.53	0.53	0.42	0.49	0.55	0.53	0.50	0.48	0.55	0.51	0.50	0.55	0.49	0.48	0.56	0.54	0.53	0.52	0.54	0.53	0.56	0.56	0.44	0.54	0.56	0.52	0.56
64	14	60	222	112	0.44	0.47	0.38	0.53	0.55	0.51	0.53	0.40	0.52	0.54	0.57	0.49	0.51	0.52	0.53	0.55	0.54	0.53	0.45	0.55	0.53	0.52	0.50	0.51	0.55	0.55	0.56	0.54	0.53	0.55	0.51	0.53
64.5	14	56	222	112	0.44	0.47	0.38	0.53	0.55	0.51	0.53	0.40	0.52	0.54	0.57	0.49	0.51	0.52	0.53	0.55	0.54	0.53	0.45	0.55	0.53	0.52	0.50	0.51	0.55	0.55	0.56	0.54	0.53	0.55	0.51	0.53
65	16	53	226	108	0.45	0.48	0.50	0.54	0.51	0.50	0.51	0.38	0.46	0.52	0.52	0.51	0.48	0.48	0.54	0.53	0.54	0.55	0.48	0.56	0.56	0.52	0.50	0.50	0.56	0.56	0.55	0.50	0.52	0.55	0.52	0.55
65.5	18	56	229	105	0.50	0.49	0.36	0.50	0.50	0.50	0.47	0.43	0.50	0.55	0.49	0.41	0.42	0.45	0.51	0.55	0.53	0.52	0.48	0.53	0.54	0.50	0.52	0.49	0.49	0.53	0.54	0.43	0.50	0.55	0.54	0.54
66	19	57	230	104	0.49	0.48	0.38	0.50	0.48	0.49	0.46	0.40	0.48	0.54	0.51	0.48	0.43	0.47	0.48	0.48	0.55	0.49	0.47	0.53	0.54	0.49	0.48	0.49	0.51	0.55	0.54	0.42	0.50	0.54	0.54	0.50
66.5	20.78	56	232	102	0.49	0.51	0.41	0.49	0.52	0.49	0.50	0.37	0.49	0.51	0.53	0.49	0.47	0.52	0.50	0.54	0.52	0.49	0.33	0.53	0.51	0.49	0.48	0.52	0.53	0.52	0.51	0.50	0.52	0.53	0.52	0.57
67	22	56	233	101	0.48	0.51	0.42	0.49	0.53	0.50	0.50	0.36	0.47	0.52	0.51	0.48	0.48	0.51	0.52	0.55	0.52	0.50	0.42	0.53	0.52	0.50	0.45	0.54	0.51	0.54	0.50	0.49	0.52	0.55	0.53	0.55
67.5	23	34	235	99	0.49	0.49	0.34	0.48	0.52	0.48	0.48	0.32	0.46	0.50	0.51	0.45	0.49	0.48	0.50	0.53	0.53	0.47	0.45	0.52	0.52	0.48	0.48	0.51	0.52	0.51	0.53	0.47	0.52	0.52	0.50	0.54
68	24	37	236	98	0.48	0.45	0.39	0.52	0.50	0.49	0.48	0.37	0.47	0.51	0.50	0.48	0.50	0.50	0.51	0.53	0.51	0.53	0.45	0.51	0.53	0.53	0.47	0.43	0.49	0.50	0.54	0.50	0.54	0.53	0.51	0.55
68.5	26	68	239	95	0.42	0.51	0.34	0.51	0.45	0.46	0.48	0.36	0.46	0.48	0.50	0.43	0.49	0.50	0.48	0.50	0.47	0.52	0.47	0.52	0.55	0.50	0.48	0.49	0.47	0.49	0.51	0.48	0.51	0.52	0.50	0.53
69	27.08	39	241	93	0.46	0.47	0.40	0.47	0.50	0.45	0.48	0.37	0.47	0.48	0.51	0.45	0.47	0.46	0.47	0.54	0.47	0.46	0.45	0.51	0.51	0.49	0.42	0.47	0.48	0.50	0.48	0.49	0.51	0.52	0.49	0.52

69.5	29	36	243	91	0.46	0.49	0.32	0.48	0.44	0.45	0.46	0.35	0.44	0.48	0.53	0.45	0.48	0.45	0.46	0.53	0.50	0.52	0.46	0.50	0.51	0.49	0.47	0.47	0.46	0.48	0.47	0.48	0.55	0.52	0.44	0.50
70	31	36	246	88	0.44	0.43	0.34	0.43	0.47	0.44	0.44	0.32	0.45	0.49	0.44	0.42	0.45	0.46	0.44	0.47	0.49	0.49	0.43	0.50	0.50	0.49	0.45	0.45	0.50	0.48	0.48	0.46	0.50	0.49	0.48	0.50
70.5	32	37	249	85	0.40	0.44	0.37	0.46	0.45	0.42	0.42	0.29	0.39	0.49	0.49	0.42	0.44	0.45	0.43	0.49	0.48	0.51	0.41	0.48	0.51	0.47	0.45	0.43	0.47	0.47	0.45	0.47	0.50	0.48	0.49	0.51
71	34	60	253	81	0.42	0.45	0.40	0.45	0.45	0.42	0.43	0.32	0.34	0.46	0.45	0.41	0.38	0.45	0.45	0.43	0.45	0.46	0.44	0.49	0.47	0.45	0.43	0.45	0.47	0.47	0.46	0.44	0.50	0.47	0.46	0.44
71.5	36	59	254	80	0.41	0.44	0.30	0.43	0.42	0.40	0.42	0.30	0.42	0.48	0.37	0.41	0.41	0.43	0.44	0.46	0.49	0.45	0.42	0.46	0.46	0.46	0.48	0.35	0.47	0.46	0.47	0.43	0.50	0.46	0.45	0.44
72	37.04	39	256	78	0.42	0.44	0.31	0.45	0.43	0.40	0.43	0.30	0.37	0.46	0.45	0.39	0.42	0.43	0.44	0.47	0.45	0.50	0.41	0.46	0.46	0.44	0.43	0.46	0.47	0.46	0.45	0.43	0.50	0.48	0.44	0.48
72.5	40	38	259	75	0.39	0.41	0.36	0.42	0.42	0.38	0.39	0.26	0.40	0.45	0.41	0.39	0.40	0.43	0.42	0.44	0.46	0.48	0.42	0.49	0.43	0.44	0.41	0.44	0.44	0.44	0.43	0.42	0.47	0.45	0.45	0.46
73	42	44	259	75	0.40	0.41	0.35	0.42	0.40	0.40	0.40	0.31	0.39	0.46	0.41	0.40	0.41	0.43	0.39	0.45	0.44	0.44	0.34	0.45	0.44	0.40	0.42	0.41	0.45	0.45	0.43	0.43	0.46	0.47	0.46	0.44
73.5	45	38	260	74	0.39	0.34	0.35	0.40	0.42	0.39	0.39	0.26	0.37	0.44	0.41	0.33	0.38	0.39	0.39	0.40	0.44	0.45	0.39	0.43	0.43	0.40	0.39	0.36	0.44	0.45	0.43	0.41	0.45	0.45	0.44	0.41
74	50.36	37	261	73	0.38	0.38	0.30	0.43	0.42	0.38	0.37	0.26	0.39	0.44	0.40	0.35	0.40	0.33	0.40	0.41	0.45	0.42	0.39	0.42	0.41	0.41	0.40	0.37	0.45	0.47	0.43	0.39	0.48	0.45	0.44	0.45
74.5	53	46	262	72	0.34	0.40	0.23	0.40	0.38	0.38	0.39	0.24	0.36	0.42	0.34	0.35	0.39	0.39	0.39	0.42	0.45	0.42	0.39	0.43	0.45	0.41	0.40	0.39	0.44	0.44	0.40	0.40	0.45	0.45	0.42	0.42
75	55	44	263	71	0.39	0.38	0.35	0.41	0.41	0.38	0.34	0.23	0.36	0.44	0.39	0.38	0.38	0.41	0.38	0.44	0.45	0.42	0.45	0.42	0.46	0.40	0.40	0.38	0.43	0.44	0.44	0.39	0.46	0.45	0.44	0.44
75.5	59	35	265	69	0.38	0.40	0.25	0.38	0.39	0.37	0.37	0.27	0.37	0.43	0.38	0.36	0.32	0.39	0.37	0.33	0.42	0.42	0.35	0.46	0.45	0.40	0.40	0.38	0.41	0.42	0.43	0.38	0.42	0.42	0.39	0.40
76	63	38	266	68	0.38	0.34	0.28	0.38	0.36	0.37	0.37	0.26	0.38	0.44	0.38	0.37	0.39	0.38	0.38	0.37	0.44	0.44	0.36	0.44	0.43	0.40	0.38	0.36	0.44	0.43	0.44	0.41	0.43	0.42	0.39	0.38
76.5	66	42	267	67	0.36	0.39	0.32	0.38	0.36	0.37	0.38	0.23	0.36	0.42	0.34	0.37	0.37	0.38	0.39	0.37	0.42	0.39	0.35	0.40	0.45	0.40	0.39	0.40	0.43	0.43	0.43	0.39	0.45	0.43	0.40	0.43
77	70	39	268	66	0.37	0.36	0.34	0.38	0.36	0.36	0.37	0.22	0.37	0.43	0.35	0.37	0.38	0.37	0.35	0.37	0.42	0.37	0.37	0.40	0.40	0.40	0.37	0.41	0.42	0.41	0.42	0.39	0.44	0.40	0.38	0.40
77.5	74	42	270	64	0.37	0.35	0.30	0.35	0.36	0.36	0.36	0.26	0.35	0.41	0.38	0.36	0.37	0.37	0.36	0.38	0.43	0.38	0.37	0.41	0.42	0.41	0.33	0.42	0.39	0.39	0.42	0.38	0.42	0.40	0.38	0.37
78	80	38	271	63	0.34	0.35	0.36	0.32	0.35	0.35	0.34	0.24	0.35	0.40	0.31	0.31	0.31	0.33	0.33	0.36	0.42	0.44	0.35	0.40	0.43	0.39	0.36	0.36	0.41	0.40	0.41	0.38	0.43	0.39	0.32	0.37
78.5	86	38	271	63	0.34	0.34	0.31	0.37	0.30	0.35	0.36	0.23	0.32	0.41	0.31	0.35	0.32	0.37	0.34	0.33	0.40	0.37	0.34	0.40	0.39	0.38	0.36	0.40	0.41	0.41	0.40	0.39	0.43	0.41	0.37	0.38
79	90.28	43	273	61	0.32	0.34	0.31	0.31	0.35	0.34	0.33	0.22	0.31	0.41	0.37	0.34	0.35	0.35	0.35	0.35	0.41	0.38	0.33	0.35	0.38	0.39	0.35	0.38	0.42	0.43	0.40	0.40	0.45	0.38	0.36	0.38
79.5	94	43	275	59	0.30	0.34	0.26	0.34	0.25	0.28	0.32	0.25	0.30	0.40	0.34	0.29	0.33	0.34	0.34	0.35	0.41	0.37	0.33	0.38	0.37	0.39	0.36	0.36	0.39	0.42	0.39	0.37	0.39	0.39	0.35	0.36
80	101.6	34	275	59	0.28	0.35	0.32	0.35	0.34	0.33	0.33	0.24	0.30	0.40	0.31	0.32	0.32	0.35	0.33	0.35	0.41	0.39	0.32	0.36	0.41	0.37	0.36	0.38	0.41	0.42	0.36	0.37	0.42	0.40	0.32	0.36
80.5	107	31	277	57	0.31	0.34	0.34	0.34	0.34	0.31	0.36	0.21	0.31	0.38	0.34	0.30	0.29	0.32	0.33	0.35	0.39	0.32	0.28	0.34	0.38	0.37	0.35	0.39	0.39	0.38	0.39	0.35	0.37	0.35	0.29	0.36
81	111.92	42	278	56	0.31	0.32	0.35	0.33	0.37	0.31	0.35	0.22	0.32	0.39	0.35	0.31	0.33	0.33	0.32	0.36	0.40	0.40	0.31	0.39	0.40	0.38	0.35	0.36	0.41	0.42	0.36	0.37	0.41	0.37	0.33	0.38
81.5	120.58	42	280	54	0.29	0.31	0.26	0.32	0.32	0.29	0.32	0.19	0.26	0.40	0.30	0.32	0.30	0.31	0.31	0.34	0.39	0.37	0.32	0.37	0.36	0.38	0.35	0.35	0.41	0.39	0.37	0.34	0.39	0.34	0.35	0.38
82	131.24	41	281	53	0.29	0.31	0.29	0.31	0.29	0.31	0.34	0.22	0.29	0.38	0.33	0.31	0.30	0.31	0.27	0.31	0.39	0.37	0.31	0.34	0.38	0.39	0.34	0.35	0.37	0.40	0.38	0.38	0.38	0.34	0.34	0.36
82.5	141	38	281	53	0.30	0.33	0.24	0.33	0.28	0.29	0.30	0.25	0.30	0.40	0.26	0.31	0.29	0.30	0.30	0.30	0.40	0.35	0.31	0.32	0.35	0.40	0.34	0.33	0.43	0.38	0.39	0.38	0.40	0.37	0.30	0.30
83	150	40	282	52	0.31	0.26	0.28	0.32	0.30	0.31	0.30	0.28	0.30	0.39	0.29	0.32	0.33	0.35	0.29	0.29	0.42	0.34	0.30	0.33	0.38	0.39	0.35	0.32	0.40	0.38	0.40	0.40	0.41	0.40	0.35	0.32
83.5	157.22	44	282	52	0.32	0.28	0.28	0.30	0.18	0.31	0.29	0.19	0.29	0.38	0.29	0.31	0.29	0.28	0.29	0.24	0.39	0.37	0.29	0.36	0.38	0.42	0.34	0.30	0.39	0.39	0.36	0.39	0.40	0.35	0.32	0.32
84	167.88	22	284	50	0.30	0.30	0.32	0.33	0.28	0.31	0.29	0.21	0.30	0.36	0.28	0.31	0.29	0.30	0.29	0.29	0.41	0.34	0.29	0.41	0.35	0.38	0.33	0.29	0.41	0.41	0.34	0.40	0.40	0.36	0.31	0.30
84.5	175.54	21	284	50	0.30	0.34	0.32	0.34	0.35	0.30	0.32	0.24	0.31	0.32	0.31	0.29	0.30	0.36	0.33	0.35	0.33	0.32	0.30	0.32	0.32	0.32	0.33	0.30	0.33	0.34	0.32	0.34	0.33	0.32	0.28	0.33
85	185.2	13	286	48	0.29	0.31	0.28	0.29	0.30	0.30	0.29	0.20	0.29	0.31	0.30	0.30	0.30	0.31	0.29	0.27	0.31	0.33	0.28	0.31	0.30	0.32	0.31	0.20	0.31	0.30	0.31	0.31	0.30	0.32	0.28	0.29

Abbreviation: COP = Cutoff Percentile; COV = Cutoff Value; RFs = Remain Features; NoLs = Number of Labels;

Table S9. Definitions of feature names.

Feature name	Definition
SpecialMedCare	Whether the patient receives specialized medical care.
CaregiverGender-Female	Primary caregiver is female.
NotHiredForeignCaregiver	No foreign caregivers employed.
CaseEligibility-Normal	Patient is a regular household case.
SpecialMedCare-PainMnagement	Patient under specialized pain management.
CaseIndependence	Hours patient can be left alone at home.
LTCEligibility-Normal	Patient eligible for standard long-term care.
ShortTermMemmory	Patient's short-term memory capacity.
CancerType-Lung	Patient diagnosed with lung cancer.
MedIntervention	Need for service intervention in medical care.
FearOfFall	Patient avoids activities due to fall fear.
HasStairs	Living environment includes stairs.
FallRisk-Bathroom	Fall risk in the bathroom.
ShoulderMobilityLimited	Limited shoulder movement.
SelfLaundry	Ability to do laundry independently.
SelfMedication	Ability to self-administer medication.
Age	Patient's age.
CoResidents-Other	Other people living with the patient.
CaregiverSleepQuality	Caregiver's sleep affected.
WoundType	Type of wound, postoperative.
WalkAbnormal	Difficulties in walking.
CaregiverStrain	Physical strain on caregiver.
PainConditionAnsweredByCaregiver	Caregiver reports on pain.
KneeMobilityLimited	Limited knee movement.
ADLScore	Score on ADL scale.
ToiletAbility	Independence in using the toilet.
BoneMedication	Bone-related medications used.
Diabetes	Patient diagnosed with diabetes.
ChildCoResidents	Children living with patient.
HavingSecondCaregiver	Presence of secondary informal caregiver.

Abbreviation: ADL = activities of daily living
